# Upregulation of an Epithelial miRNA Is Associated with Immune Evasion in Progressive Bronchial Premalignant Lesions

**DOI:** 10.1158/2326-6066.CIR-25-0431

**Published:** 2026-02-11

**Authors:** Boting Ning, Darren J. Chiu, Roxana M. Pfefferkorn, Erin Cullinane, Yohana Kefella, Erin Kane, Valerie Reyes-Ortiz, Gang Liu, Xiaohui Zhang, Hanqiao Liu, Lila Sultan, Emily Green, Myrtha Constant, Avrum E. Spira, Joshua D. Campbell, Mary E. Reid, Xaralabos Varelas, Eric J. Burks, Marc E. Lenburg, Sarah A. Mazzilli, Jennifer E. Beane

**Affiliations:** 1 Department of Medicine, Boston University Chobanian & Avedisian School of Medicine, Boston, Massachusetts.; 2 Department of Pathology and Laboratory Medicine, Boston University Chobanian & Avedisian School of Medicine, Boston, Massachusetts.; 3 Department of Medicine, Roswell Park Comprehensive Cancer Center, Buffalo, New York.; 4 Department of Biochemistry, Boston University Chobanian & Avedisian School of Medicine, Boston, Massachusetts.

## Abstract

Bronchial premalignant lesions (PML), precursors of lung squamous cell carcinoma, have distinct molecular subtypes. The proliferative subtype, enriched with bronchial dysplasia, had decreased expression of an antigen-processing/presentation gene coexpression module in progressive/persistent versus regressive PMLs, suggesting a functional impact of these genes on immune evasion. In this study, we performed miRNA sequencing, miRNA *in situ* hybridization, and spatial proteomics of bronchial biopsies from patients at high risk for lung cancer. An miRNA–gene network analysis identified hsa-miR-149-5p as a potential regulator of the antigen presentation gene module. Staining on adjacent biopsy tissue showed that hsa-miR-149-5p was predominantly expressed in the epithelium and upregulated in progressive/persistent proliferative lesions. Targets of this miRNA, the transcriptional coactivator of MHC-I gene expression, *NLRC5*, and the genes it regulates, were downregulated in these lesions. Decreased *NLRC5* expression reduced both IFNγ-induced MHC-I surface expression and CD8 T-cell cytotoxicity in lung squamous cancer cells. In PMLs, basal cells with high levels of NLRC5 were in close spatial proximity to CD8 T cells, suggesting that these cells exhibit increased functional MHC-I gene expression *in vivo*. These findings indicate a functional role for hsa-miR-149-5p in PML progression/persistence and suggest this axis as a potential therapeutic target for PML immunomodulation.

## Teaser

Using integrative analysis of bronchial premalignant lesions, the authors identify hsa-miR-149-5p as a potential regulator of immune evasion in progressive/persistent lesions and a candidate therapeutic target for intercepting disease progression.

## Introduction

Lung squamous cell carcinoma (LUSC) arises in the bronchial airway and makes up approximately 25% of all lung cancers, the leading cause of cancer-related deaths in the United States and worldwide (1, 2). LUSC is thought to progress from bronchial premalignant lesions (PML) that are characterized by aberrant epithelial architecture and abnormal expansion of airway basal cells. The pathologic grading of PMLs has been well documented (3) and describes progressive epithelial changes, including hyperplasia, metaplasia, dysplasia (mild, moderate, and severe), and carcinoma *in situ* (CIS). As the normal bronchial epithelium is damaged, the differentiated pseudostratified columnar epithelium becomes hyperplastic with the proliferation of basal like cells, or metaplastic with the expansion of squamous cells, and worsens to dysplasia and CIS with increased cytologic aberrations, including nuclear pleomorphism, increased mitotic figures, and altered cellular differentiation (3–6). PMLs that progress from normal, hyperplasia, or metaplasia to a high-grade PML (moderate/severe dysplasia or CIS) or high-grade PMLs that persist over time are associated with a higher risk for lung cancer development at the site of the PML or elsewhere in the lung (4, 7, 8). Only a small fraction of high-grade PMLs, however, progress to CIS or invasive cancer and more than half regress to a lower histologic grade spontaneously without any intervention (4, 5). DNA and methylation profiling of CIS epithelium revealed increased chromosomal instability and somatic mutations in lesions that progress to LUSC but that spontaneous regression of CIS can occur in cases with known cancer driver mutations (9). These results suggest that alterations in immune surveillance may be a key determinant of progression to invasive carcinoma.

To gain insights into progression mechanisms, our group and others have profiled the transcriptomes of PMLs biopsied from patients at high risk for developing lung cancer over time and observed that PML progression is associated with suppression of immune surveillance via multiple mechanisms, including impairment of antigen processing and presentation and IFNγ signaling, increased expression of immune checkpoints and cytokines inhibiting T-cell activation, and by promoting the recruitment of myeloid-derived tumor suppressor cells (6, 10–12). Our studies identified a transcriptionally distinct group of PMLs (“proliferative subtype”), based on nine gene coexpression modules, which expresses high levels of cell cycle and proliferation genes, is enriched with bronchial dysplasia, and has high basal cell and low ciliated cell composition. Persistent or progressive lesions transcriptionally classified as proliferative-subtype PMLs have reduced numbers of CD8 T cells and decreased expression of a coexpression module involved in interferon signaling and antigen processing and presentation, termed the antigen presentation gene module, compared with regressive PMLs. Despite the multiple reports of immune evasion in progressive high-grade lesions, little is known about what drives immune-related gene regulation.

In the absence of T-cell exhaustion in our cohort, we hypothesized that miRNA may be regulating the expression of genes in the antigen presentation gene module associated with PML progression. miRNAs are short noncoding RNAs that suppress gene expression and facilitate transcript degradation through complementary base-pair binding to the 3′ untranslated region of gene transcripts (13). One study profiling 14 miRNAs observed hsa-miR-34c to be associated with PML histologic changes (14); however, miRNA expression alterations contributing to bronchial PML progression have not been reported. To address this question, we used sample-matched mRNA and miRNA sequencing (miRNA-seq) data from longitudinally collected PML biopsies to probe whether miRNA-mediated gene regulation leads to PML progression associated with an immunosuppressive microenvironment. We identified hsa-miR-149-5p as a potential regulator of the antigen presentation gene module associated with proliferative-subtype lesion progression. We found hsa-miR-149-5p to be highly expressed in the epithelium, upregulated in progressing/persistent compared with regressing proliferative-subtype PMLs, and a regulator of MHC-I and antigen-processing genes via *NLRC5* that influences recruitment of CD8 T cells. Our results suggest that hsa-miR-149-5p may be a potential therapeutic target for preventing PML progression to lung cancer.

## Materials and Methods

### Sample collection

**Figure 1.**
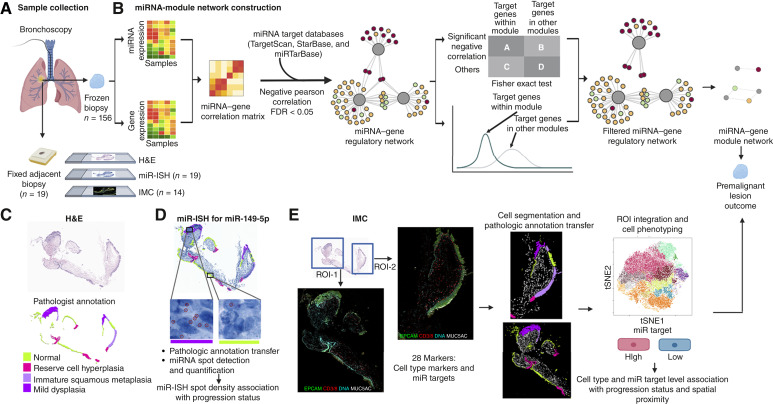
Study overview. **A,** High-quality miRNA-seq data was generated from 156 frozen endobronchial biopsies (*n* = 28 patients). These samples also had paired mRNA sequencing data. Among the 156 biopsies, 19 adjacent FFPE biopsies, transcriptionally defined as the proliferative molecular subtype, were sectioned and stained to assess spatial localization of key findings. **B,** The paired miRNA–mRNA sequencing data were used to construct a miRNA–gene network based on database evidence of miRNA target genes and significant negative correlation between miRNA and gene expression patterns (gray circles are miRNAs and red, green, and yellow circles are genes for which the color represents their membership in previously described gene coexpression modules that define PML molecular subtype). Target information was obtained by combining a sequence-based prediction database and experimentally validated databases. Pearson correlation was calculated from expression residuals for each miRNA–mRNA pair, and those with significant negative Pearson correlation coefficients (FDR ≤ 0.05) were selected. Next, we filtered edges to identify miRNAs that may have central gene expression regulatory roles within the miRNA–gene network in each gene module. For each gene module, we identified and retained only the connections from miRNAs for which (i) there are more significantly negative correlated target genes in the module than in other modules (Fisher exact test, odds ratio>1) and (ii) the Fisher-Z–transformed Pearson correlation coefficient densities with its targets within the module were more negative than its targets in other modules (*t* test, t < 0). miRNAs with significant results from both tests (FDR ≤ 0.05) were considered module-associated miRNAs, and other miRNAs were removed from the network. **C–E,** For each FFPE biopsy, three consecutive sections were stained for different purposes: (**C**) H&E to annotate the histologic grades present in the epithelial tissue, (**D**) chromogenic miRNA-ISH to quantify hsa-miR-149-5p expression and localization, and (**E**) IMC using 28 metal-tagged antibodies to identify epithelial and immune cell types and hsa-miR-149-5p target protein expression and localization. Single or multiple ROIs per sample were selected for IMC ablation in 14 samples. The epithelial histology in both miRNA-ISH and IMC images was annotated based on the corresponding adjacent H&E section. The schematic was constructed using data images from Fig. 4. [Created in BioRender. Chiu, D. (2026) https://BioRender.com/g78j632]

The patient enrollment and sample collection procedures were described previously (10). The study was conducted with approval from the Roswell Park Comprehensive Cancer Center and the Boston University School of Medicine Institutional Review Boards, which adhere to the ethical principles and guidelines delineated in the Belmont Report. All subjects provided written informed consent. Briefly, endobronchial biopsies and bronchial brushings were collected via autofluorescence bronchoscopy from high-risk individuals in a low-dose CT lung cancer screening program at approximately 1-year intervals. Biopsy outcome status was defined as previously described (10), in which the outcome status of each biopsy (progressive/persistent, regressive, normal stable, or unknown) was based on the worst histology recorded at the same anatomic location in future procedures. During the bronchoscopy, two biopsy samples were obtained from each sampled lung anatomic location. One biopsy was formalin fixed and paraffin embedded (FFPE) and used for histologic evaluation, miRNA *in situ* hybridization (miR-ISH), and image mass cytometry (IMC) assays, and the other biopsy was frozen and used for RNA isolation and mRNA and miRNA-seq. Bronchial brushes were collected at each bronchoscopy from a normal-appearing area of the right or left mainstem bronchus. In our prior study (10), patients were divided into a discovery (*n* = 197 biopsies, *n* = 91 brushes, and *n* = 30 patients) and validation set (*n* = 111 biopsies, *n* = 49 brushes, and *n* = 20 patients) based on the time of initial sample collection. In this study, discovery cohort samples with sufficient miRNA underwent miRNA library preparation and sequencing (167 biopsies and 91 brushes and *n* = 30 patients). Additional adjacent fixed samples (19 biopsies and *n* = 9 patients) were subject to detailed pathologic annotation on hematoxylin and eosin (H&E) stained slides, miR-ISH, and IMC assays (Fig. 1A–E).

### miRNA library preparation, sequencing, and data preprocessing

miRNA was extracted from biopsies or brushing samples using the miRNeasy Mini Kit (Qiagen) according to the manufacturer’s instructions (10). miRNA-seq libraries were prepared using NEBNext Multiplex Small RNA Library Prep Set for Illumina (New England Biolabs). Sequencing adapters that target the 3′ hydroxyl group of small RNAs were ligated and the transcripts were reverse transcribed and PCR amplified into single-stranded cDNA libraries. The libraries were pooled and size selected for small RNA fragments on PAGE gel in groups of 6 to 10. The libraries were sequenced on Illumina HiSeq 2500 to generate more than 10 million single-read 35 to 36 nucleotide (nt) reads per sample (15).

Demultiplexing and generation of FASTQ files were performed using Illumina CASAVA v1.8.2. FastQC v0.11.7 (RRID: SCR_014583) was used to examine the quality of raw reads and cutadapt v1.18 was used for trimming the sequencing adapters (5′- AGA​TCG​GAA​GAG​CAC​ACG​TCT​GAA​CTC​CAG​TCA​C-3′, -trim-n; refs. 16, 17). Reads unlikely to be properly sequenced from mature miRNA transcripts were discarded (reads shorter than 16 nt or longer than 25 nt with –m 16 –M 25 in cutadapt). Alignment and quantification of mature miRNAs based on miRBase v22 (RRID: SCR_003152) were performed with miRDeep2 v0.1.0 (18, 19) with mapper.pl parameters –e –h –m –i –j and quantifier.pl parameter –k –j –d -P. For mature miRNAs associated with multiple precursor miRNAs, the max counts were used.

We performed miRNA-seq sample quality assessments and miRNA filtering separately for each sample type (endobronchial biopsies and bronchial brushes). We limited the analysis to the miRNA samples with previously analyzed [GSE109743 and Database of Genotypes and Phenotypes (dbGaP):phs003185.v1.p1] matched mRNA expression profiles (*n* = 160 biopsies and *n* = 89 brushes; ref. 10). The raw counts were converted into log_2_ counts per million (CPM) of miRNA reads mapped (log CPM) and quantile normalized with voom (20). miRNAs with log CPM less than 1 in more than half of the samples were removed. We excluded samples (*n* = 4 biopsies and *n* = 2 brushes) if sequencing quality metrics were outside two SDs from the mean. The quality metrics we used included the first and the second principal components from the principal component analysis using filtered miRNA expression, the mean Pearson expression correlation with all other samples across the filtered miRNAs, and the transcript integrity number (TIN) of the matched mRNA sequencing sample [calculated using RSeQC v3.0.0 (21), RRID: SCR_005275]. Finally, 156 biopsies and 87 brush samples from 30 patients were retained. The gene filter and normalization described above were performed on the final high-quality samples with matching mRNA data (*n* = 525 miRNAs in biopsies and *n* = 489 miRNAs in brushes). The residual miRNA expressions adjusting for sequencing batch were computed with limma (RRID: SCR_010943; ref. 22) for downstream analysis.

### miRNA–gene network construction to identify miRNAs associated with gene coexpression modules that define PML molecular subtypes

To capture the association of miRNAs with the previously described (10) nine co-expression gene modules within biopsy samples, we first constructed a miRNA–gene regulatory network focused on the module genes (*n* = 3,936; Supplementary Table S1; ref. 23). For this network, we utilized both the predicted gene targets for an miRNA and the correlation between an miRNA and its targets (Fig. 1B). The predicted gene targets for each miRNA were defined as those identified by any of the three miRNA target databases, including TargetScan v7.2, starBase, and miRTarBase v7.0 (24–26). Between each miRNA and its predicted target gene, the Pearson correlation coefficient was calculated using the residual expression values, of miRNA (described above) and gene (adjusted for median TIN and batch as in Beane and colleagues; ref. 10), and the significance level was adjusted using FDR. Only the edges with significant and negative Pearson correlation coefficients (FDR ≤ 0.05) between miRNA and predicted target genes were retained in the network.

**Figure 2.**
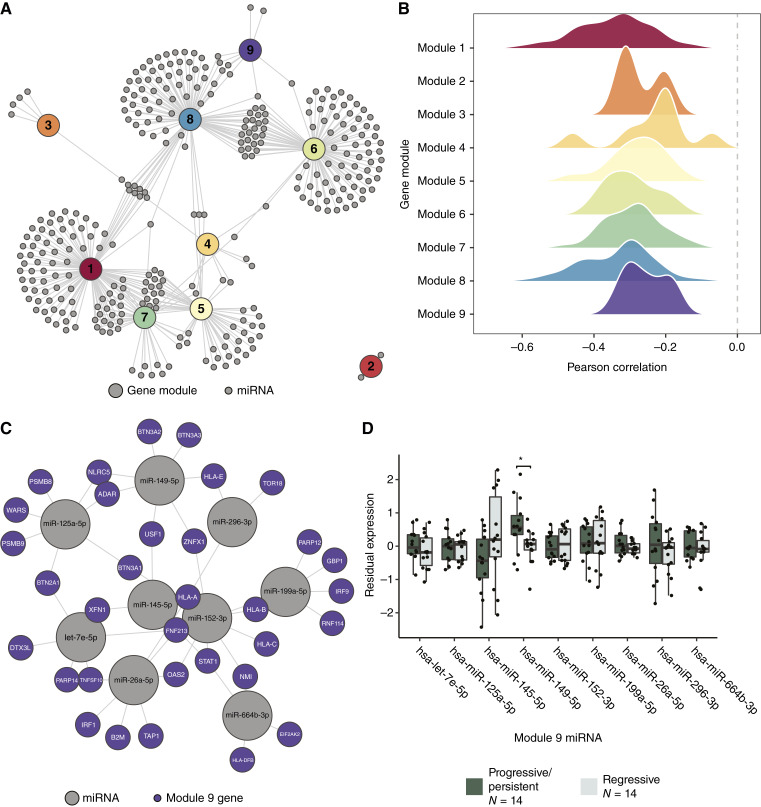
miRNA–gene network analysis identifies miRNAs associated with the antigen presentation gene module downregulated in progressive proliferative subtype PMLs. **A,** miRNA–gene module network plot shows 321 miRNAs connected to nine gene modules, in which gray circles are miRNAs and colored circles are the gene modules. Edges reflect a miRNA that may have strong suppressive regulatory role over the predicted target genes within the connected gene module based on the two statistical test filters. **B,** Pearson correlation densities between the module-associated miRNAs connected to each gene module in the miRNA–gene module network and the corresponding gene module GSVA score. **C,** miRNA–gene network plot of miRNAs connected to the antigen presentation gene module [module 9, purple circle in (**A**)]. Edges show the significant negative correlations between the miRNAs (gray circles) and the predicted target genes (purple circles). **D,** Boxplots of residual expression levels of the nine miRNAs specifically connected to module 9 in proliferative subtype biopsies stratified by their outcome status (progressive/persistent samples in dark green and regressive samples in light green). *, *P* < 0.05, determined by a linear mixed-effects model.

After constructing the miRNA–gene regulatory network, we sought to retain only connections in which miRNAs had regulatory relationships with a gene module that were stronger and more specific than with other gene modules (Fig. 1B). For each miRNA, we first performed Fisher-Z transformation on the Pearson correlation coefficient density of a miRNA with its targets and compared the density with its targets within the module with the density with targets in other modules using a two-sample or one-sample (if only one target is within a group) *t* test to select miRNAs with strong negative relationships to modules. We also used Fisher exact tests to determine whether a miRNA had more significantly negatively correlated target genes in the module of interest than in other modules (odds ratio > 1). The results of both tests were adjusted using FDR and miRNAs, with significant results for the same gene module from both tests (FDR ≤ 0.05) identified as specifically regulating that module. Any connection between a miRNA and predicted target genes of a module that did not pass both tests was removed. The final network can be viewed as a filtered subset of the full miRNA–gene regulatory network that captures the potential regulatory relationship between miRNAs and gene modules (Fig. 2A). Each miRNA in the network was assigned to its associated one or more modules (module-associated miRNA). Summary scores were computed based on miRNAs connected to each gene module (27) using gene set variation analysis (GSVA).

### Identification of miRNAs and genes associated with PML progression status

The association between miRNAs that may regulate gene modules and PML progression status was determined based on whether the expression level of an miRNA is significantly different between the progressive/persistent and the regressive PMLs of the proliferative subtype (*P* < 0.05). Within each model, miRNA residual expressions were used as the dependent variable, PML progression status was the main independent variable, and patient was included as a random effect using the “duplicateCorrelation()” function from limma v3.44.3 (22).

To identify the genes that are associated with PML progression status, we used the full gene expression data of the discovery and validation cohorts from our previous publication, including those samples without matched miRNA-seq data (10). Similar linear mixed-effect models were performed with gene residual expressions (adjusted for sequencing batch and TIN) as the dependent variable and PML progression status as the independent variable while adjusting patient as a random effect. We further validated gene associations with PML progression status using an external microarray dataset (GSE114489; ref. 12), in which linear models were used with probe intensities as the dependent variable and progression status as the main independent variable. The progressive/persistent group included the persistent bronchial dysplasia and progressive nondysplasia samples based on the original annotation, and the regressive group included the regressive bronchial dysplasia group.

The association between hsa-miR-149-5p and the antigen presentation gene module was examined in three datasets with sample-matched miRNA and gene expression profiles, including the airway brushing samples from patients with PMLs, The Cancer Genome Atlas (TCGA) LUSC samples, and Airway Epithelial Gene Expression in the Diagnosis of Lung Cancer (AEGIS) samples. Gene set enrichment analysis (GSEA; ref. 28) was performed with the R package fgsea (RRID: SCR_003199) with random seed of 1,234 and nperm = 10,000, in which genes were ranked by their Pearson correlation coefficient with hsa-miR-149-5p. The negative correlation between hsa-miR-149-5p and NLRC5 was also validated in these three datasets using Pearson correlation. To evaluate the expression levels of hsa-miR-149-5p and its target genes between tumor and adjacent benign tissue, we utilized miRNA (*n* = 45 paired samples) and gene (*n* = 51 paired samples) expression profiles of TCGA LUSC tumor tissue that had matched adjacent benign tissue. Linear models using limma- and voom-transformed data (20, 22) were performed to derive the genes differentially expressed between tumor and benign tissue using the tissue type as the main variable while controlling for patient.

### Examination of the cell type–specific expression of hsa-miR-149-5p

To examine whether an miRNA is potentially specifically expressed within a certain cell type, we examined the correlation between the expression level of the miRNA and cell-type marker genes. The method for calculating Pearson correlation coefficients has been described in the previous section. The following cell type marker genes were used: *CD3G* for T cells, *CD19* for B cells, *CD68* for macrophages, *KRT5* for basal cells, *FOXJ1* for ciliated cells, *MUC5AC* for goblet epithelial cells, *SCGB1A1* for club cells, *MUC5B* for mucus-secretory cells, and *CEACAM5* for peri-goblet cells (29). Also, miRNA expression data from functional annotation of the mammalian genome 5 (FANTOM5) project were used to evaluate whether the expression of a miRNA is enriched within a certain group of samples (30). Classification of FANTOM5 samples to cell types was based on cell ontology annotation from FANTOM5. Samples belonging to the “epithelial cell” or “leukocyte” group were used as the epithelial and immune compartment. Samples were ranked by their normalized expression levels of hsa-miR-149-5p and GSEA (28) using the R package fgsea (RRID: SCR_003199) with seed 1,234 and nperm = 10,000 to test whether samples with higher hsa-miR-149-5p expression were enriched for samples from a specific group of cell types. Gene expression data from FANTOM5 were also used to examine the correlation between hsa-miR-149-5p and *NLRC5* among different cell types. The visualization of the miRNA expression by samples was done in similar fashion as in fantom.gsc.riken.jp.

### *In vitro* perturbation of hsa-miR-149-5p and *NLRC5* expression

Human LUSC cells, SW900 (ATCC, cat. #HTB-59, RRID: CVCL_1731), were grown in RPMI medium and transfected using Lipofectamine RNAiMAX (Thermo Fisher Scientific, cat. #13778075) following the manufacturer’s protocol. Cells were transfected with (i) hsa-miR-149-5p (*mir*Vana, Thermo Fisher Scientific, ref. #4464066, ID: MC12788); (ii) miR scramble (mock) control (MISSION, Sigma, cat. #HMC0002); and (iii) siNLRC5 (NLRC5 84166 siRNA, SMARTpool, Dharmacon, cat. #L-018267-020010). Cells were incubated in transfection mix for 48 hours, washed, and lysed in QIAzol (Qiagen cat. #79306) for 5 minutes at room temperature before being stored at −80°C until RNA isolation. Following RNA isolation, qRT-PCR (6–10 replicates per condition) was conducted using 1 ng of RNA per sample that was reverse transcribed using the Transcriptor First Strand cDNA Synthesis Kit (Roche, cat. #04896866001) following the supplier’s protocol. For miRNA quantification, 1 ng total RNA was used in cDNA synthesis via the TaqMan Advanced miRNA cDNA Synthesis Kit (Thermo Fisher Scientific, cat. #A28007). qRT-PCR was performed using the TaqManTM Fast Advanced Master Mix (Applied Biosystems, cat. #4444557) and TaqMan probes (Thermo Fisher Scientific) *PPIA* (Hs04194521_s1), *NLRC5* (Hs01072123_m1), *HLA-A* (Hs01058806_g1), *HLA-B* (Hs00818803_g1), *HLA-C* (Hs00740298_g1), *PSMB9* (Hs00160610_m1), *TAP1* (Hs00388675_m1), hsa-miR-149-5p (477917_mir), and hsa-miR-98-5p (478590_mir; Supplementary Table S2). The 2^−ΔΔCT^ method (31) was used for relative transcript quantification. *PPIA* was used as the reference gene for mRNA quantification. For miRNA quantification, miR98 served as an internal control. The average ΔCT of mock controls in each 96-well qPCR plate (representing experiment batch) was used as the reference sample. Gene expression differences between treatment conditions were determined by a two-sided Wilcoxon test.

### Quantification of MHC-I cell surface expression

SW900 cells were transfected as described above with siNLRC5 or mock control. Following 24 hours of transfection, cells were stimulated with IFNγ (Recombinant Human IFNγ Protein, R&D Systems, cat. #285-IF-100) at a concentration of 80 ng/mL. Following 24 hours of stimulation, cells were harvested for qRT-PCR validation of the NLRC5 knockdown (∼70,000 cells) and for flow cytometry (∼500,000 cells) to measure MHC-I surface expression. Cells for flow cytometry were stained on ice for 30 minutes with anti–human HLA-A, HLA-B, and HLA-C (pan-MHC-I, clone W6/32, PE, BioLegend, cat. # 311406, RRID: AB_314875, 1:500) in PBS/4% FBS/2 mmol/L of EDTA after Fc block (BioLegend, cat. #422301, RRID: AB_2818986; 1:50, 5 minutes). A viability dye (Zombie Aqua, BioLegend, cat. #423101; 1:800) was included. Samples were washed and acquired by the Cytek Aurora flow cytometer using standard tube mode, followed by analysis using FlowJo v.10 software (RRID: SCR_008520). MCH-I levels in live cells were analyzed using a linear mixed model with group as a fixed factor (mock, mock/IFNγ, siNLRC5, and siNLRC5/IFNγ) and sample as random effect. Significance was assessed with the contrast (mock/IFNγ–mock) – (siNLRC5/IFNγ–siNLRC5) to test whether INFγ-induced change in the controls exceeded that in the NLRC5 knockdown using lmerTest v3.1-3 (RRID: SCR_015656; ref. 32) and emmeans v1.10.3 (RRID: SCR_018734; ref. 33).

### Preparation of T cells

T cells were isolated and expanded from peripheral blood mononuclear cells (STEMCELL Technologies, cat. #70025.1) by culturing cells in RPMI supplemented with 10% FBS (Gibco, Thermo Fisher Scientific, cat. #A5670801), 1% penicillin–streptomycin (Thermo Fisher Scientific, cat. #15140-122), and 100 U/mL of IL2 (Thermo Fisher Scientific, cat. #200-02-10UG) with the addition of anti–human CD3 (clone OKT3, Thermo Fisher Scientific, cat. #16-0037-81, RRID: AB_468854; 1 μg/mL in 2 mL) and anti–human CD28 (clone CD28.2, Thermo Fisher Scientific, cat. #16-0289-81, RRID: AB_468854; 5 μg/mL) for 7 days and then without the CD3 and CD28 for an additional 7 days. The expansion of T cells was confirmed using flow cytometry, and staining of cells was performed as described above with anti-CD45 (clone HI30, Pacific Blue, BioLegend, cat. #304022, RRID: AB_493655; 1:200) and anti-CD8 (clone SK1, PerCP, BioLegend, cat. #344708, RRID: AB_1967149; 1:100).

### Cytotoxic T-lymphocyte killing assay

SW900 cells were plated in 96-well plates at a density of 10,000 cells per well and transfected as described above with siNLRC5 or mock control. After 24 hours of transfection, cells were stimulated with IFNγ (Recombinant Human IFNγ Protein, R&D Systems) at a concentration of 80 ng/mL. Following 24 hours of stimulation, T cells were expanded as indicated and were plated with the SW900 cells at a ratio of 1 (SW900):5 (T cell). After 16 hours of incubation, the amount of lactate dehydrogenase (LDH) released was assessed using the CyQUANT LDH Cytotoxicity Assay (Thermo Fisher Scientific, cat. #C20300) according to the manufacturer’s protocol. Wells containing only SW900 cells were used as (i) low-level controls (LDH spontaneous activity) and (ii) high-level controls with the addition of lysis buffer (LDH maximum release). The percentage of cell-mediated cytotoxicity was calculated based on the formula [LDH absorbance value (AV) – LDH spontaneous activity AV]/(LDH maximum activity AV – LDH spontaneous activity AV) × 100. For each condition, siNLRC5, or mock control, the mean LDH spontaneous activity AV (*n* = 6) and mean LDH maximum activity AV (*n* = 12) were used in the formula. The percentage of cell-mediated cytotoxicity was compared between cells transfected with siNLRC5 (*n* = 18) or mock control (*n* = 18) via a two-sided *t* test.

### Biopsy selection, sectioning, staining, and pathologist annotation of digitized images

FFPE tissue sections from 19 proliferative biopsies from nine patients (*n* = 10 progressive/persistent and *n* = 9 regressive) were selected from samples for which adjacent biopsies were profiled by mRNA-seq and miRNA-seq (Supplementary Table S3). Three sequential tissue sections were stained using H&E for annotation of histopathology features, chromogenic miRNA-ISH (using miRNAscope, ACD Bio, cat. #324530) with a hsa-miR-149-5p probe (ACD Bio, cat. #1058711-S1) to assess hsa-miR-149-5p expression, and IMC using a cocktail of 28 antibodies targeted to epithelial and immune populations, as well as NLRC5 and its downstream targets (for 14 of the 19 biopsies). The H&Es were scanned at 40X using the Leica Aperio AT2 scanner and the miRNA-ISH slides were scanned under oil immersion at 63X using the Leica Aperio Versa Scanner. The digitized H&E image files were uploaded to the deepPath PixelView software (https://www.deeppath.ai) and the epithelial tissue was annotated by the pathologist (E.J.B.) by creating masks for the following: normal epithelium, reserve cell hyperplasia, goblet cell hyperplasia, immature squamous metaplasia, squamous metaplasia, and mild, moderate, or severe squamous dysplasia. These annotations were manually transferred to digitized images of the miRNA-ISH and IMC using QuPath v0.3.2 software (RRID: SCR_018257; ref. 34) and reviewed for accuracy by the pathologist.

### Quantification of hsa-miR-149-5p expression in biopsies via miRNAscope

For miRNA-ISH, 5-μm FFPE tissue sections were stained using the miRNAscope HD Assay Red (ACD Bio, cat. #324530) with probe SR-hsa-miR-149-5p-S1 (ACD Bio, cat. #1058711-S1). The staining was performed using the manufacturer’s protocol; however, treatment with Protease III was reduced to 10 minutes to conserve tissue morphology of the small biopsy samples.

A pixel classifier to detect the spots of hsa-miR-149-5p signal was trained on a representative region (250,000 μm^2^) selected in each sample using the artificial neural network model from QuPath v0.3.2 with default multiscale features of three channels—hematoxylin, probe stain, and residual channels (34). To validate the classifier performance, we selected a representative region (16,726 ± 4,332 mm^2^) different from the training regions in each sample and annotated the hsa-miR-149-5p spots manually. The recall [calculated as true positive (TP) divided by TP plus false negative], the precision (calculated as TP divided by TP plus false positive), and the F1 score (calculated as the harmonic mean of recall and precision) were calculated per sample. The hsa-miR-149-5p density is defined either as hsa-miR-149-5p–predicted spots per annotated area or as hsa-miR-149-5p–predicted spots per cell. The cell number was estimated by nuclei segmentation using StarDist extension v0.3.2 (35) with the pretrained brightfield model implemented via QuPath software in regions annotated as epithelium. The association of hsa-miR-149-5p density with progression status was modeled as a linear mixed-effect model, with the interaction of progression status and histology as fixed effect and sample ID as random effect using lmerTest v3.1-3 (32). To evaluate the association of hsa-miR-149-5p density with histology, a linear mixed-effect model with histology as fixed effect and sample ID as random effect was applied to the miRNA-ISH images with progressive/persistent lesions or regressive lesions, respectively.

### IMC staining and ablation

The IMC experiments assayed the levels of 28 proteins and DNA (Supplementary Table S4). Antibodies were preconjugated (Standard BioTools) or conjugated to lanthanide isotopes following the MaxPar Antibody Labeling Kit as per the manufacturer’s protocol. The IMC staining was done using the proximal level of the 14 FFPE tissue sample slides analyzed via miRNAscope assay (Supplementary Table S3). The slides were dewaxed in xylene for 10 minutes, followed by two more xylene washes of 5 minutes each. Tissue was rehydrated in sequential alcohol dilutions of 100% (three consecutive washes), 95% (two consecutive washes), 80%, and 70% for 10 minutes each before washing in deionized water for 10 minutes. Heat-induced epitope retrieval was performed in Tris-EDTA pH 8.5 antigen retrieval buffer containing 5% glycerol and heating to 96°C in the microwave for 30 minutes. Slides were allowed to cool and washed twice in deionized water and then twice in PBS (10 minutes each). Samples were blocked with 3% BSA in PBS for 45 minutes at room temperature in a wet chamber. The antibody cocktail was prepared to a final concentration of 0.5% BSA and added to the samples for overnight incubation at 4°C. The stained samples were washed twice in 0.2% Triton X-100/PBS, followed by one wash in PBS for 8 minutes each. A 1:1,000 dilution of Iridium intercalators (Standard BioTools) in PBS was used to stain DNA for 30 minutes at room temperature. Finally, slides were washed in PBS for 8 minutes and deionized water for 5 minutes before air drying. Regions of interest (ROI) were laser ablated using the Hyperion Imaging Mass Cytometry system (RRID: SCR_023195) with a 200 Hz frequency at 1 μm^2^ resolution.

### IMC data processing and quality control

The IMC data were segmented into single cells, and single-cell data were extracted using the Steinbock (v0.14.1) toolkit (36). Briefly, the raw data (in MCD format) were converted to standardized OME-TIFF files and single-cell masks were generated using the TissueNet model in Cellpose (37). DNA1 and DNA2 were used as the nuclei channel, and multiple markers, including KRT5, KRT8, E-cadherin, SCGB1A1, MUC5AC, MUC5B, FOXJ1, CEACAM5, CD3, CD4, CD8A, CD20, CD68, HLA-DR, αSMA, and vimentin (VIM), were used as the membrane/cytosol channel. Single-cell features, including mean intensity of each marker and spatial coordinates, were extracted from the single-cell masks across all available channels. The extracted single-cell data were converted to a SpatialExperiment object using the imcRtools v1.8.0 R package (RRID: SCR_027613; ref. 36) for downstream analysis. ROIs less than 1,000 cells were removed from this analysis (*n* = 7 ROIs) and cells with an area less than first percentile (21 μm^2^) or larger than 98th percentile (198 μm^2^) were filtered out (*n* = 2,804 cells). A total of 87,401 cells from 24 ROIs were used for final analysis. Antibodies with poor signal or nonspecific staining (HLA-ABC, Ki67, and CD25) were excluded from the analysis.

### IMC data clustering and differential composition analysis

The mean intensity of the single-cell raw measurement for each marker was log transformed and *z*-score normalized with respect to each ROI, referred to as log-Z normalization. The canonical phenotypic markers for epithelial cells (KRT5, KRT8, p40, E-cadherin, MUC5AC, MUC5B, CEACAM5, SCGB1A1, and FOXJ1), immune cells (CD20, CD3, CD4, CD8A, GZMB, PDL1, CD68, CD163, and HLA-DR), and stromal cells (αSMA and VIM) were used for Uniform Manifold Approximation and Projection (UMAP) dimension reduction, fastMNN batch correction (RRID: SCR_017351; ref. 38), and PhenoGraph clustering (RRID: SCR_022603; ref. 39) of all the cells. Broad cell types, including epithelial, immune, and stromal cells, were annotated based on the expression of KRT5, E-cadherin, CD20, CD3, HLA-DR, αSMA, and VIM. Given that the proportions of epithelial and nonepithelial regions are different across ROIs, we analyzed the epithelial and nonepithelial (including broad immune and stromal cells) regions separately. For each region type (epithelial or nonepithelial), signals were log-Z normalized within each ROI and used for t-distributed stochastic neighbor embedding (tSNE) dimension reduction and PhenoGraph clustering (39). In the nonepithelium compartment, a cluster was identified in which more than half of the cells had epithelial histology annotations. A total of 1,260 cells with any epithelial histology annotation in this cluster, which were shown to localize in epithelium in IMC images, were reclassified as broad epithelial cells. Then, we repeated the clustering analysis as described above. Cell types in epithelial regions were identified based on epithelial and immune cell markers, whereas nonepithelial cell types were identified based on immune and stromal cell markers. The differential composition analysis of the cell types by histology, smoking, and progression status was performed using sccomp v.1.7.14 (40) within each region type.

### Quantification of NLRC5 protein expression and its association with tissue and sample characteristics

We observed that the NLRC5 levels were significantly higher for cells within epithelium compared with the nonepithelial regions. To compare the NLRC5 levels across ROIs, we resampled the cells in nonepithelial regions to equalize the proportions of cells between epithelial and nonepithelial regions in each ROI, followed by *z*-score normalization of the log-transformed NLRC5 intensities, referred to as log-Z stromal-normalized NLRC5. Epithelial cells were dichotomized as NLRC5 high or NLRC5 low using the overall average of the log-Z stromal-normalized NLRC5 across all epithelial cells as the cutoff. The association of log-Z stromal-normalized NLRC5 expression with progression status was modeled using a linear mixed-effect model, with the interaction of progression status and histology as fixed effect and ROI as random effect using lmerTest v3.1-3 (32). The estimated marginal means for the fitted model were computed by emmeans v1.10.3 (33).

### IMC spatial cell–cell proximity analysis

Cell neighbors for each cell were identified by distance-based expansion with a centroid distance threshold of 30 mm using the buildSpatialGraph() function in imcRtools v1.8.0 (36). Immune cell type annotations from both epithelial and nonepithelial regions were merged for the same cell types. Broad epithelial or epithelial subtypes were stratified by the NLRC5 level as previously described. Spatial cell–cell proximity analysis for each ROI was performed using a standard neighborhood permutation test (41), with 500 iterations using the testInteractions() function in imcRtools v1.8.0 (36). The spatial proximity score was defined as the fraction of perturbations having proximity counts equal to or less than the observed counts for each pair of cell type entries. Difference in spatial proximity between NLRC5-high and NLRC5-low epithelial cells or epithelial subtypes with respect to immune subtypes was assessed by the two-sided paired Wilcoxon test.

## Results

### miRNA–gene network analysis identifies miRNAs associated with gene coexpression modules that define PML molecular subtypes

As miRNAs have been shown to be important in carcinogenesis (42, 43), we sought to construct a miRNA–gene network from bronchial PMLs to elucidate how miRNAs may be negatively regulating target genes associated with PML progression. To construct a miRNA–gene network, we generated high-quality miRNA-seq data from *n* = 156 endobronchial biopsies from 28 patients who had matching bulk RNA sequencing data (Fig. 1A; ref. 10). This subset was representative of the larger previously published cohort (10), based on the observed associations between lesion molecular subtype, smoking status, and histology (*χ*^2^ test *P* < 0.05; Table 1; Supplementary Table S6). The paired miRNA and mRNA expression data were used to construct the network in which nodes are miRNAs and genes and edges represent significant negative correlations between miRNAs and their predicted target genes. After removing miRNA/gene nodes without any connections, the miRNA–gene network consisted of 14,744 edges between 464 miRNAs and 2,296 genes. The median number of connected target genes for each miRNA was 18. Next, we filtered edges (Fig. 1B; see “Materials and Methods”) to identify module-associated miRNAs that may have central gene expression regulatory roles within the miRNA–gene network in each of the nine previously described (10) gene modules that define the PML molecular subtypes (Supplementary Table S1). For each gene module, we identified and retained only the connections from miRNAs for which associations with the module target genes are stronger (more negative) than with non-module target genes and for which the significant negatively correlated target genes are significantly enriched within the module. After excluding nodes without any connections and summarizing miRNA–gene connections to the gene module level, we obtained a bipartite directed network consisting of 321 miRNA nodes targeting genes in the nine gene modules (Fig. 2A; Supplementary Table S7). The connectivity of the miRNA–gene module network did not show a dependence on the number of genes in a module and 94 miRNAs had connections to more than one gene module.

**Table 1.** Clinical annotations for endobronchial biopsies that were profiled by miRNA-seq stratified by PML molecular subtypes.

​	Proliferative (*n* = 46)	Inflammatory (*n* = 27)	Secretory (*n* = 46)	Normal (*n* = 37)	​
Dysplasia grade	​	​	​	​	chi = 38.50, *P* = 0.0033
Normal	3 (6.5)	4 (14.8)	12 (26.1)	10 (27)	
Hyperplasia	4 (8.7)	8 (29.6)	7 (15.2)	6 (16.2)	
Metaplasia	8 (17.4)	8 (39.6)	9 (19.6)	12 (32.4)	
Mild dysplasia	5 (10.9)	1 (3.7)	8 (17.4)	2 (5.4)	
Moderate dysplasia	19 (41.3)	4 (14.8)	7 (15.2)	5 (13.5)	
Severe dysplasia	7 (15.2)	1 (3.7)	1 (2.2)	1 (2.7)	
Unknown	0 (0)	1 (3.7)	2 (4.4)	1 (2.7)	
Smoking status (genomic prediction)	40 (87)	11 (40.7)	35 (76.1)	12 (32.4)	chi = 35.20, *P* < 0.001
Progression status	​	​	​	​	chi = 24.37, *P* = 0.0038
Progression/persistent	14 (30.4)	4 (14.8)	14 (30.4)	5 (13.6)	
Regressing	14 (30.4)	3 (11.1)	5 (10.9)	3 (8.1)	
Normal/stable	7 (15.2)	12 (44.4)	8 (17.4)	14 (37.8)	
Unknown	11 (23.9)	8 (29.6)	19 (41.3)	15 (40.5)	

Percentages are reported for categorical clinical variables, and statistical tests were conducted using *χ*^2^ tests.

To validate that our network captures biologically relevant miRNA–gene relationships, for each module, we confirmed that the correlation density between the expression levels of module-associated miRNAs and a module metagene score computed via GSVA was left-shifted from zero (Fig. 2B). We also observed a significant negative correlation pattern between the GSVA score of a gene module and the GSVA score calculated from the module-associated miRNAs (i.e., negative correlation along the diagonal line in Supplementary Fig. S1; FDR <0.05, Pearson correlation), except for module 2. Moreover, the connections between miRNA and gene modules in the network reflected multifaceted functions of miRNAs demonstrated by previous studies (44, 45). For example, miR-200a/b-3p was connected to gene modules with genes associated with the extracellular matrix and the immune activation (module 1 and 8; Supplementary Table S7; refs. 46, 47). These results suggest that our miRNA–gene module network reveals biologically meaningful regulatory relationships between miRNAs and the predicted target genes.

### Genes in the PML progression–associated antigen presentation module are connected to hsa-miR-149-5p

The antigen presentation gene module (module 9), enriched with genes associated with interferon signaling and antigen presentation and processing, has previously (10) been shown to be downregulated among proliferative subtype progressive/persistent compared with the regressive PMLs. To identify miRNAs that potentially contribute to the PML progressive phenotype, we used the miRNA–gene module network to identify nine miRNAs (hsa-let-7e-5p, hsa-miR-125a-5p, hsa-miR-145-5p, hsa-miR-149-5p, hsa-miR-152-3p, hsa-miR-199a-5p, hsa-miR-26a-5p, hsa-miR-296-3p, and hsa-miR-664b-3p) that were significantly negatively correlated with 33 of the 112 module 9 genes (Fig. 2C). For each of these nine miRNAs, the miRNA–gene correlations with predicted target genes in module 9 were significantly more negative than correlations with predicted target genes in the other gene modules, and the significant negatively correlated target genes were enriched within module 9 (FDR <0.05, *t* test and Fisher exact test; Supplementary Table S8). Next, we examined whether the expression levels of these miRNAs were associated with the progressive status of proliferative subtype PMLs. hsa-miR-149-5p was the only significantly upregulated miRNA in progressive/persistent (*n* = 14) versus regressive (*n* = 14) proliferative subtype PMLs (*P* < 0.05, linear mixed-effect model; Fig. 2D; Supplementary Table S9), suggesting that its higher expression is associated with worse lesion prognosis in concordance with its previously reported oncogenic role in different cancer settings (48).

### hsa-miR-149-5p targets MHC-I genes by suppressing NLRC5 expression

**Figure 3.**
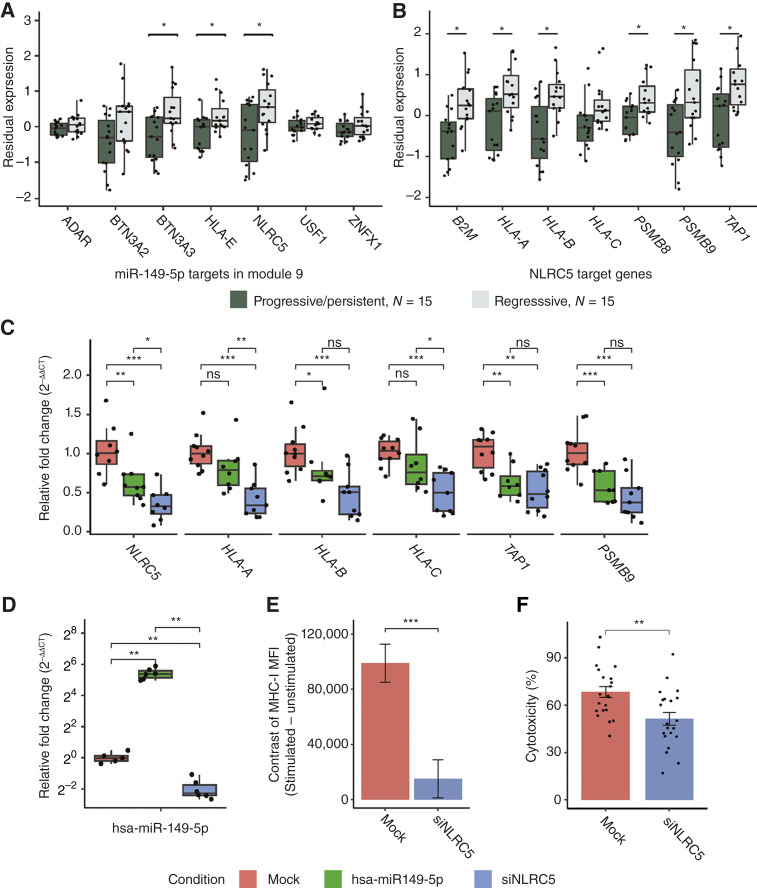
Hsa-miR-149-5p and *NLRC5* target genes are associated with PML progression, and *in vitro* modulation of *NLRC5* changes MHC-1 expression and CD8 T cell–mediated cytotoxicity. **A** and **B**, Boxplots of residual expression levels of progressive/persistent (dark green) and regressive (light green) proliferative subtype PMLs from the discovery cohort (GSE109473) for (**A**) hsa-miR-149-5p significant negatively correlated target genes in module 9 and (**B**) *NLRC5* target genes. **C** and **D**, SW900 cells were transfected with mock control (red orange), hsa-miR-149-5p (green), or siNLRC5 (blue) for 48 hours. Relative gene expression detected by qRT-PCR (6–10 replicates per condition) of (**C**) *NLRC5* and its downstream targets (*HLA-A*, *HLA-B*, *HLA-C*, *TAP1*, and *PSMB9*) and (**D**) hsa-miR-149-5p. *PPIA* was used as the reference gene to calculate relative fold change values. **E **and** F,** Barplot showing the difference in (**E**) mean fluorescent intensity (MFI) of surface MHC-I and (**F**) percent cytotoxicity between IFNγ-simulated and unstimulated conditions between mock- and siNLRC5-treated SW900 cells. Data indicate median with IQR, and whiskers indicate minimum and maximum measurement. Error bars indicate standard errors. *P* values were determined using mixed-effect linear models (**A**, **B**, and **E**), two-sided Wilcoxon tests (**C** and **D**), and two-sided *t* test (**F**). *, *P* ≤ 0.05; **, *P* ≤ 0.01; ***, *P* ≤ 0.001; ns, no significance.

Next, we sought to elucidate how hsa-miR-149-5p upregulation contributes to lesion progression by investigating the seven genes in module 9 connected to the miRNA in the network analysis (Fig. 2C). We compared the expression levels of these genes between the proliferative subtype progressive/persistent (*n* = 15) versus the regressive (*n* = 15) PMLs from the discovery cohort in our prior work (GSE109473; ref. 10). All seven targets were downregulated in the progressive/persistent PMLs, and the expression level of three target genes, *BTN3A3*, *HLA-E*, and *NLRC5*, was significantly lower in the progressive/persistent proliferative PMLs than in the regressive ones (*P* < 0.05, linear mixed-effect model; Fig. 3A; Supplementary Table S10). The lower expression of these genes among PMLs that progress was also observed in the proliferative PMLs from our validation cohort (*n* = 7 progressive/persistent and *n* = 12 regressive) and in an independent dataset from Merrick and colleagues (*n* = 32 progressive/persistent and *n* = 15 regressive, GSE114489; Supplementary Table S10).

To further validate the association between hsa-miR-149-5p and module 9, we leveraged three airway and lung tumor datasets with sample-matched miRNA and gene expression profiles: airway brushing samples (*n* = 87) obtained during the same bronchoscopy procedure as the endobronchial biopsies used to derive the miRNA–RNA network cohort, TCGA LUSC resection samples (*n* = 475; ref. 49), and airway brushings from the AEGIS trials (*n* = 341; refs. 50, 51). Module 9 genes were significantly enriched among genes that were negatively correlated with hsa-miR-149-5p in all datasets (*P* < 0.001, GSEA; Supplementary Fig. S2A–S2D). Also, 47.7% of the genes in the leading edge driving the signal were overlapping across four studies, including four of the hsa-miR-149-5p–predicted target genes in module 9 (*BTN3A2*, *BTN3A3*, *HLA-E*, and *NLRC5*; Supplementary Fig. S2E). These observations suggest that hsa-miR-149-5p may promote the progressive PML phenotype by specifically suppressing target genes in the antigen presentation gene module.

Among hsa-miR-149-5p target genes that were significantly downregulated in progressive/persistent lesions, *NLRC5* was notable because prior studies have shown that it is a member of the NOD-like receptor (NLR) family and functions as a transcriptional activator of the MHC-I genes (52). *NLRC5* is a predicted target for hsa-miR-149-5p in two of the three miRNA target databases (24–26) used in our network construction and was negatively correlated with hsa-miR-149-5p in the three airway and lung tissue datasets (*P* ≤ 0.05, Pearson correlation; Supplementary Fig. S3A–S3C). Additionally, in TCGA LUSC tumor tissue with matched adjacent benign tissue, the expression of hsa-miR-149-5p was upregulated and expression of NLRC5 was downregulated in tumor compared with adjacent benign tissue (*P* < 0.001, linear mixed-effect model; Supplementary Fig. S4A and S4B).

We previously reported that depletion of CD8 T cells is associated with PML progression (10) and hypothesized that this could partially be due to hsa-miR-149-5p repressing the expression of the MHC-I genes by downregulating *NLRC5* expression. Seven genes (*B2M*, *HLA-A*, *HLA-B*, *HLA-C*, *PSMB8*, *PSMB9*, and *TAP1*) were identified to be directly regulated by NLRC5 based on literature reviews (53, 54). NLRC5 promoter binding at the mouse orthologues of these genes can be observed via chromatin immunoprecipitation sequencing experiments from mouse T cells (55). The seven *NLRC5* target genes are also within module 9, and the expression levels for all, except *HLA-C*, were significantly downregulated within the proliferative subtype progressive/persistent versus regressive PMLs in the previously published discovery cohort (Fig. 3B; Supplementary Table S11). We further validated the downregulation of *NLRC5* target genes in progressive/persistent versus regressive PMLs in the proliferative subtype PMLs from our validation cohort and in the samples from Merrick and colleagues (GSE114489; Supplementary Table S11).

qRT-PCR of SW900, lung squamous cancer cell line, transfected with hsa-miR-149-5p, siNLRC5, or a miR scramble control (mock) sequence demonstrated that cells transfected with hsa-miR-149-5p significantly increased hsa-miR-149-5p and decreased *NLRC5* and its target genes, *HLA-B*, *TAP1*, and *PSMB9*, compared with mock. Transfection with siNLRC5 significantly decreased *NLRC5* and its target genes *HLA-A/B/C*, *PSMB9*, and *TAP1* (*P* < 0.05, two-sided Wilcoxon test; Fig. 3C and D). The experiments also suggest a potential negative feedback loop between *NLRC5* and hsa-miR-149-5p as *NLRC5* was decreased more strongly by experiments with siNLRC5 than hsa-miR-149-5p alone and siNLRC5 significantly decreased hsa-miR-149-5p expression compared with mock (*P* < 0.01, two-sided Wilcoxon test; Fig. 3C). The computational and qRT-PCR results suggest that hsa-miR-149-5p may promote lesion progression by suppressing *NLRC5* and MHC-I gene expression.

To assess the functional consequences of *NLRC5* knockdown, using flow cytometry, we measured MHC-I surface expression in SW900 cells transfected with siNLRC5 or a mock sequence with and without IFNγ stimulation. Decreased *NLRC5* expression (Supplementary Fig. S5A) resulted in a significant reduction in MHC-I surface expression upon stimulation with IFNγ compared with mock control (*P* < 0.001, linear mixed-effect model; Fig. 3E; Supplementary Fig. S5B). Based on these results, we next examined whether SW900 cells with decreased *NLRC5* expression had reduced T cell–mediated killing. Following the coculture of T cells and SW900 cells, there was a significant reduction in cell-mediated cytotoxicity in IFNγ-stimulated siNLRC5 SW900s compared with mock control (*P* < 0.01, two-sided *t* test; Fig. 3F). These data indicate that decreased *NLRC5* expression reduces both MHC-I surface expression and CD8 T-cell cytotoxicity, suggesting that decreased *NLRC5* expression may facilitate immune evasion in progressive/persistent lesions.

### hsa-miR-149-5p expression is enriched within epithelial cells

**Figure 4.**
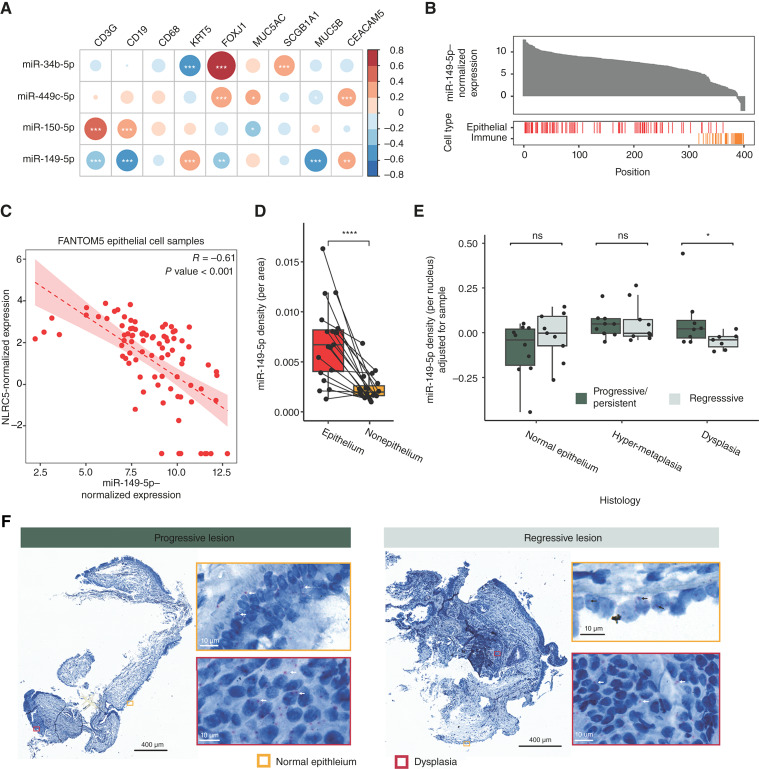
hsa-miR-149-5p is highly expressed within the epithelium and associates with PML progression. **A,** Bubble plot showing the correlation between the residual expression level of hsa-miR-149-5p and cell type marker genes, including *CD3G* (T cells), *CD19* (B cells), *CD68* (macrophages), *KRT5* (basal cells), *FOXJ1* (ciliated cells), *MUC5AC* (goblet cells), *SCGB1A1* (club cells), *MUC5B* (secretory cells), and *CEACAM5* (peri-goblet cells). Correlation results for hsa-miR-34b-5p, hsa-miR-449c-5p, and hsa-miR-150-5p are shown as controls. **B,** Enrichment of hsa-miR-149-5p across cell type compartments in the FANTOM5 project. Bar chart shows the normalized expression levels of hsa-miR-149-5p in the FANTOM5 samples (*n* = 399) (top). Vertical bars indicate the position of FANTOM5 samples derived from the epithelial (red) or immune (orange) cell compartments (bottom). **C,** Scatter plot of the Pearson correlation between the normalized expression levels of hsa-miR-149-5p and *NLRC5* within the FANTOM5 samples derived from the epithelial cell compartment (*n* = 87). The dashed red line represents the linear regression fit and the shaded region indicates the 95% confidence interval. **D–F,** The hsa-miR-149-5p density was detected by miRNA-ISH from proliferative-subtype PMLs (*n* = 19; 10 progressive/persistent and nine regressive). **D,** Boxplot showing the hsa-miR-149-5p density per area between the epithelium and nonepithelium regions. **E,** hsa-miR-149-5p density per nucleus between the progressive/persistent and regressive PMLs within regions of normal epithelium, regions with hyperplasia or metaplasia (hyper-metaplasia), and regions of bronchial dysplasia. **F,** Representative miRNA-ISH staining of hsa-miR-149-5p for progressive/persistent PMLs (top) and regressive PMLs (bottom). Arrows indicate stained hsa-miR-149-5p within epithelium. Boxplots indicate median with IQR and whiskers indicate minimum and maximum measurement in (**D**) and (**E**). *P* values were FDR (Benjamini and Hochberg) adjusted and determined by Pearson correlation coefficients (**A**), the two-sided paired Wilcoxon test (**D**), and a linear mixed-effects model (**E**). *, *P* ≤ 0.05; **, *P* ≤ 0.01; ***, *P* ≤ 0.001; ****, *P* ≤ 0.0001; ns, no significance.

Interpreting the potential role of hsa-miR-149-5p in lesion progression is dependent on the cell type specificity of its expression and its targets. To identify the cell type specificity of hsa-miR-149-5p expression, we examined the correlation pattern between hsa-miR-149-5p and canonical cell type markers within our lesion biopsy samples. We observed significant positive correlations between hsa-miR-149-5p and the basal cell marker, *KRT5* (FDR <0.001, Pearson correlation; ref. 56), and the peri-goblet cell marker, *CEACAM5* (FDR <0.01, Pearson correlation; Fig. 4A; ref. 29). In comparison, hsa-miR-34b-5p and hsa-miR-449c-5p, which are expressed highly in mucociliary epithelia, were positively correlated with *FOXJ1*, and hematopoiesis-essential hsa-miR-150-5p was positively correlated with B- and T-cell markers (Fig. 4A; ref. 57). We also utilized cell type–specific transcriptomic data from the FANTOM5 project (30) and ranked samples based on the normalized expression level of hsa-miR-149-5p and identified whether samples derived from epithelial or lymphoid cell types were enriched among samples with high hsa-miR-149-5p expression. Samples from epithelial cells (*n* = 87) were positively enriched among samples with high hsa-miR-149-5p expression (*P* < 0.001, GSEA), whereas samples from immune cells (*n* = 37) were negatively enriched (*P* < 0.01, GSEA; Fig. 4B). Additionally, the expression of hsa-miR-149-5p and *NLRC5* in the FANTOM5 data was significantly negatively correlated between samples from epithelial cells (*P* < 0.01, Pearson correlation; Fig. 4C), but not in samples from immune cells (Supplementary Fig. S6), suggesting that the suppression of *NLRC5* by hsa-miR-149-5p may be specific to epithelial cells. The highly enriched expression of hsa-miR-149-5p and negative regulation relationship between hsa-miR-149-5p and *NLRC5* within the epithelial cells suggested that hsa-miR-149-5p may be involved in epithelial cell–mediated immune evasion in early lung cancer.

### Spatial localization of hsa-miR-149-5p in PMLs showed higher expression in the epithelium that was associated with PML progression

To localize and validate the computational findings, consecutive tissue sections from proliferative subtype endobronchial biopsies underwent staining with H&E to annotate the histologic grades present in the epithelium and chromogenic miRNA-ISH with an hsa-miR-149-5p probe to measure hsa-miR-149-5p expression and localization (Fig. 1A, C, and D; Supplementary Table S3). An expert pathologist annotated the histologic grades of the epithelium in each digitized whole-slide H&E image, and these annotations were transferred to the miRNA-ISH and grouped as follows: (i) normal; (ii) hyperplasia, immature squamous metaplasia, and squamous metaplasia (denoted as hyper-metaplasia); and (iii) squamous dysplasia. The worst histologic grade observed in the H&E matched the clinical diagnosis in 81% of biopsies, suggesting that many features in the clinical specimens were retained in subsequent sections (Supplementary Tables S3 and S12). A pixel classifier was trained to detect hsa-miR-149-5p signal spots in the miRNA-ISH data and its performance was validated on regions (16,726 ± 4,332 mm^2^) containing manual spot annotations for each sample. The classifier showed high recall (0.84 ± 0.06), precision (0.75 ± 0.14), and F1 (0.78 ± 0.08) measurement that was not associated with lesion outcomes (Supplementary Fig. S7A and S7B). The detected hsa-miR-149-5p spots per area were higher in the epithelium compared with nonepithelium (*P* < 0.001, two-sided paired Wilcoxon test; Fig. 4D), supporting the computational results showing high hsa-miR-149-5p expression correlation with epithelial markers. We also observed higher hsa-miR-149-5p spot density in epithelial areas with squamous dysplasia in the progressive/persistent compared with regressive PMLs (*P* = 0.05, linear mixed-effect model; Fig. 4E and F). This difference was likely driven by changes in progressive/persistent PMLs as we observed that hsa-miR-149-5p spot density in dysplastic regions was increased compared with normal epithelium in the progressive/persistent lesions but not in regressive lesions (*P* < 0.05, linear mixed-effect model; Supplementary Fig. S8A and S8B). These results confirm that hsa-miR-149-5p is expressed more highly in epithelial cells and that expression differences observed between progressive/persistent versus regressive PMLs may occur within regions of bronchial dysplasia.

### IMC data show differential cell composition associated with histology and correlated protein expression between NLRC5 and its downstream targets

**Figure 5.**
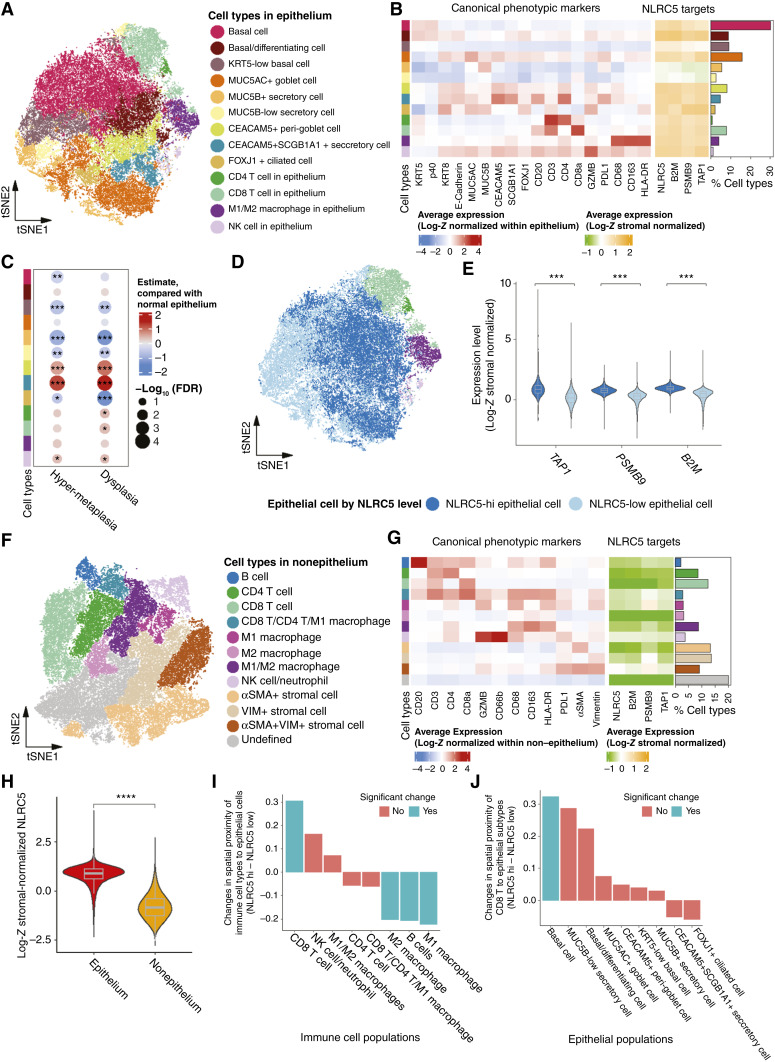
Protein expression of NLRC5 and its targets is correlated, and basal cells with increased NLRC5 expression are in close spatial proximity to CD8 T cells. **A,** tSNE visualization of eight epithelial and four immune cell populations within the epithelium (*n* = 41,147 cells) from IMC data (*n* = 14 samples; seven progressive and seven regressive). **B,** Heatmap of the average log-Z–normalized expression within the epithelium of canonical markers (left) and the average log-Z stromal-normalized expression within the epithelium of NLRC5 and its downstream targets (right) in identified cell types from (**A**). Bar plot shows the relative proportion of each cell type. **C,** Bubble plot shows the differential composition of cell types in hyper-metaplasia and dysplasia as compared with normal epithelium. Color represents the compositional estimate, and dot size represents the log FDR value computed by sccomp. **D,** tSNE visualization of epithelial cell populations colored by NLRC5-high (dark blue) vs. NLRC5-low (light blue) expression. **E,** Violin plot of the log-Z stromal-normalized expression of NLRC5 target genes (*TAP1*, *PSMB9*, and *B2M*) stratified by the NLRC5-high and NLRC5-low labels. **F,** tSNE visualization of eight immune and three stromal cell population in the stroma region (*n* = 46,254 cells) from IMC data (*n* = 14 samples; seven progressive and seven regressive). **G,** Heatmap of the average log-Z–normalized expression within the stroma of canonical markers (left) and the average log-Z stromal-normalized expression within the epithelium of NLRC5 and its downstream targets (right) in identified cell types from (**F**). Bar plot shows the relative proportion of each cell type. **H,** Violin plot of log-Z stromal-normalized expression of NLRC5 in the epithelium (red) vs. the stroma or nonepithelial tissue (yellow). **I** and **J,** Cellular neighbors for each cell were defined based on a centroid distance within 30 μm. Cell–cell proximity score within each ROI (*n* = 24) was computed by the permutation test in imcRtools. Cyan bar represents *P* value < 0.05 whereas orange bars are not significant. **I,** Bar plot showing the change in spatial proximity of immune cell populations to NLRC5-high vs. NLRC5-low epithelial cells, averaged across all ROIs. **J,** Bar plot showing the change in spatial proximity of CD8 T cells to NLRC5-high vs. NLRC5-low epithelial populations, averaged across all ROIs. *P* values were determined by the sccomp differential composition test (**C**), the two-sided unpaired Wilcoxon test (**E** and **H**), and the two-sided paired Wilcoxon test (**I** and **J**). *, *P* ≤ 0.05; **, *P* ≤ 0.01; ***, *P* ≤ 0.001.

The transcriptomic and miRNA-ISH data suggest that hsa-miR-149-5p is more highly expressed in the epithelium, so we sought to confirm that the protein expression between NLRC5 and its downstream targets was correlated within epithelial cells via IMC. Using consecutive tissue sections from those used in the miRNA-ISH experiment, we ablated ROIs containing epithelial and nonepithelial tissue, and cells from these regions were separated using the expression of broad cell type markers (KRT5, E-cadherin, CD20, CD3, HLA-DR, αSMA, and VIM) coupled with pathologist annotation described above (Fig. 1E; Supplementary Fig. S9A and S9B). The 41,147 high-quality cells from the epithelial-predominant tissue regions were used to identify 13 cell clusters (Fig. 5A) containing epithelial and immune cell types based on the expression of canonical cell markers (Fig. 5B). Each cell cluster had cells from at least 13 samples (of 14 samples tested). We did not, however, detect significant associations between cell cluster abundance and smoking status (FDR >0.05, sccomp differential composition test, Supplementary Fig. S10A and S10B), potentially because of the underrepresentation of former smokers. The expected location of cell types in the IMC images helped confirm the cluster labels. For example, clusters labeled as basal cells were found close to the basement membrane whereas clusters labeled as fully differentiated cells (i.e., MUC5AC^+^ goblet cells) were found at the apical surface in both normal and hyper-metaplastic regions (Supplementary Fig. S12A). Differential composition analysis showed that fully differentiated cells, including MUC5B^+^ secretory cells and FOXJ1^+^ ciliated cells, were decreased in regions of hyper-metaplasia and dysplasia, whereas intermediate-state cells, including CEACAM5^+^ peri-goblet cells and CEACAM5^+^SCGB1A1^+^ secretory cells, were increased (FDR <0.001, sccomp differential composition test, Fig. 5C; Supplementary Fig. S10C). The infiltration of immune cells, including CD8 T cells, CD4 T cells, and NK cells, was significantly increased in dysplastic regions (FDR <0.001, sccomp differential composition test, Fig. 5C; Supplementary Fig. S10C).

The epithelial cells were dichotomized into cells with high or low NLRC5 expression based on the average of the log *z*-score stromal-normalized NLRC5 expression across ROIs (Fig. 5D; Supplementary Fig. S10D and S10E). The expression of NLRC5 downstream targets (B2M, PSMB9, and TAP1) was significantly lower in NLRC5-low versus NLRC5-high cells (*P* < 0.001, two-sided Wilcoxon test, Fig. 5E) and NLRC5 and its downstream targets were significantly correlated across epithelial cells (r > 0.6, *P* < 0.001, Supplementary Fig. S10F). The proportion of NLRC5-low epithelial cells was enriched among KRT5-low basal cells, MUC5B-low secretory cells, and MUC5B^+^ secretory cells, whereas the proportion of NLRC5-high epithelial cells was enriched among basal cells, differentiating basal cells, CEACAM5^+^ peri-goblets cells, CEACAM5^+^SCGB1A1^+^ secretory cells, and FOXJ1^+^ ciliated cells (FDR <0.05, sccomp differential composition test, Supplementary Fig. S10G). These results suggest that protein expression of NLRC5 and three of its downstream targets is correlated in the epithelium and that NLRC5-high epithelial cells are enriched in intermediate-state cell populations (e.g., CEACAM5^+^ peri-goblets cells and CEACAM5^+^SCGB1A1^+^ secretory cells) that are increased in abundance in hyper-metaplastic and dysplastic regions.

### Basal cells with high NLRC5 protein expression were in closer spatial proximity to CD8 T cells

Next, we wanted to assess the spatial proximity of immune cell populations to epithelial cells with NLRC5-high versus -low protein expression. Toward this goal, we clustered the 46,254 high-quality cells in the nonepithelial tissue regions to identify 12 cell clusters (Fig. 5F) based on the expression of canonical cell markers (Fig. 5G). Each cell cluster had cells from all samples, and we did not observe significant associations between cell cluster abundance and smoking status (Supplementary Fig. S11A and S11B). NLRC5 expression was significantly lower in the nonepithelial tissue regions compared with the epithelial regions (*P* < 0.001, Wilcoxon test, Fig. 5H), suggesting that NLRC5 is more active within the epithelium. Given that some immune cells in nonepithelium regions are located near the epithelial basement membranes, we perform spatial proximity analysis using both epithelial and nonepithelial region immune cells. We tested the spatial cell–cell proximity of NLRC5-high and NLRC5-low epithelial cells to immune cells using the neighborhood permutation test (cell neighbors defined by a centroid distance = 30 μm, with *n* = 500 permutations). We observed that NLRC5-high epithelial cells had closer spatial proximity to CD8 T cells whereas NLRC5-low epithelial cells had closer spatial proximity to B cells and macrophages (*P* = 0.05, two-sided paired Wilcoxon test, Fig. 5I; Supplementary Fig. S12B and S12C). Among the epithelial cell types, NLRC5-high basal cells had significantly closer spatial proximity to CD8 T cells than NLRC5-low basal cells (*P* < 0.05, two-sided paired Wilcoxon test, Fig. 5J; Supplementary Figs. S11C, S12B, and S12C). The observation that NLRC5-high basal cells are near CD8 T cells suggests that these cells may be important mediators of a CD8 T-cell response to dysplastic lesion development.

### Basal cells in progressive versus regressive PMLs have decreased NLRC5 expression in hyper-metaplasia and dysplasia regions

**Figure 6.**
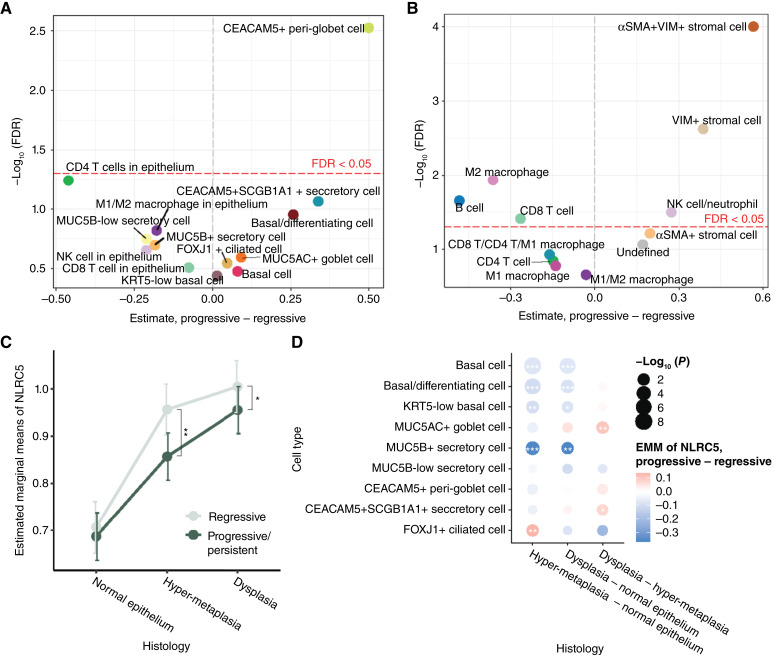
Immune and stroma cell composition and epithelial NLRC5 expression is associated with PML progression. **A** and **B**, Volcano plots showing the differential composition of progressive/persistent (*n* = 7) compared with regressive (*n* = 7) PMLs for (**A**) cell populations identified in epithelium and (**B**) cell populations identified in stroma regions. **C,** Line plot showing the estimated marginal mean of NLRC5 expression with SD in epithelial cells of progressive/persistent (dark green) and regressive (light green) PMLs, respectively, across the three histology regions. Although histology groups were modeled as categorical, the line plot is used to facilitate visualization of trends across histology grades. **D,** Bubble plot showing the differential expression of NLRC5 between progressive/persistent and regressive PMLs in each epithelial cell population across the three histology contrasts. Color represents the estimate, and dot size represents the log *P* value computed by a linear mixed-effects model. *P* values were determined by the sccomp differential composition test (**A** and **B**) and linear mixed-effects models (**C** and **D**). *, *P* ≤ 0.05; **, *P* ≤ 0.01; ***, *P* ≤ 0.001.

Given that NLRC5-high basal cells are near CD8 T cells, we wanted to test whether there were cell type compositional differences between progressive/persistent and regressive lesions as previously we have shown the reduction of CD8 T cells in progressive lesions (10). Differential cell composition analysis showed that the proportion of CEACAM5^+^ peri-goblet cells was significantly higher in the epithelial tissue regions of progressive/persistent lesions compared with regressive lesions (FDR <0.01, sccomp differential composition test, Fig. 6A; Supplementary Fig. S10H). In the nonepithelial tissue regions, CD8 T cells, B cells and M2 (CD163^+^ only) macrophages were decreased, whereas VIM^+^ stromal cells and αSMA^+^VIM^+^ stromal cells were increased in progressive/persistent lesions compared with regressive lesions (FDR <0.05, sccomp differential composition test, Fig. 6B; Supplementary Fig. S11D). Basal cells were not decreased in abundance in progressive/persistent lesions, so we next investigated differences in NLRC5 expression. We found that NLRC5 protein levels were increased in epithelial regions of hyperplasia, metaplasia, and dysplasia compared with normal epithelium (*P* < 0.001, linear mixed-effect model, Fig. 6C) in regressive compared with progression/persistent lesions. As NLRC5 increases with increasing histologic severity, but increases less in progressive/persistent lesions, we wanted to test the interaction effect between histology and progression status within the epithelial cell types. We found that NLRC5 expression in the basal cells, basal/differentiating cells, KRT5-low basal cells, and MUC5B^+^ secretory cells was decreased in progressive/persistent lesions compared with regressive lesions in the hyper-metaplasia and dysplasia regions (*P* < 0.05, linear mixed-effect model, Fig. 6D; Supplementary Fig. S12B and S12C). These analyses suggest that NLRC5 expression is decreased within basal cells in progressive/persistent compared with regressive PMLs and thus may be associated with the observed depletion of CD8 T cells.

## Discussion

To move from treating to preventing lung cancer, a detailed understanding of the mechanisms that contribute to the development and progression of bronchial PMLs is needed. Our group and others have profiled the transcriptomes of bronchial PMLs, precursor lesions of LUSC, and showed that immune evasion is associated with increased histologic severity and lesion progression (6, 10–12, 58). We previously demonstrated that bronchial PMLs can be classified into four distinct molecular subtypes based on nine conserved gene coexpression modules. The proliferative molecular subtype was significantly enriched with bronchial dysplasia, and within this subtype, decreased expression of genes involved in antigen processing and presentation (module 9) and decreased immune cell infiltration were associated with persistent/progressive versus regressive PMLs (10). In related prior work, progressive bronchial dysplasia was associated with decreases in T cells and HLA-DRA^+^ epithelial cells (12). In progressive CIS lesions, genetic and epigenetic alterations in antigen presentation–related genes, including loss of heterozygosity in the HLA region, have been observed (11). These findings support the hypothesis that impaired antigen processing and presentation contribute to lesion progression. However, the transcriptional regulators contributing to the dysregulation, especially in non-CIS PMLs, are still poorly understood. In this study, we measured miRNA expression profiles from longitudinally collected endobronchial biopsies previously profiled by mRNA sequencing and constructed an miRNA–mRNA module network to identify miRNAs associated with the antigen presentation gene co-expression module (module 9). We found that hsa-miR-149-5p, a miRNA highly expressed in the epithelium, was upregulated among the persistent/progressive compared with regressive proliferative PMLs and repressed *NLRC5*, the transcriptional regulator of MHC-I genes. We found that decreased *NLRC5* in an LUSC cell line blunted IFNγ-induced MHC-I surface expression and reduced CD8 T-cell cytotoxicity. Furthermore, we used miR-ISH and IMC to characterize the spatial localization of hsa-miR-149-5p, NLRC5, and its downstream targets in the context of the lesion cellular architecture and the immune microenvironment in biopsy tissue sections. Our results demonstrate that NLRC5 and the MHC-I genes that it regulates are increased with increasing histologic severity in the epithelium. However, when comparing progressive/persistent versus regressive proliferative subtype PMLs within different histologic grades, there is a decrease in the expression of NLRC5 and its downstream targets partially driven by increased hsa-miR-149-5p expression. Decreased NLRC5 in basal cells was associated with reduced spatial proximity to CD8 T cells and may contribute to the immune evasion phenotype.

Among the direct targets of hsa-miR-149-5p within module 9 was *NLRC5*, a gene that regulates the expression of MHC-I (*HLA-A*, *HLA-B*, *HLA-C*, and *B2M*) and immunoproteasome genes (*PSMB8*, *PSMB9*, and *TAP1*). miR-ISH analysis indicated that hsa-miR-149-5p was expressed in epithelial cells of PMLs and *in vitro* experiments showed that *NLRC5* and several of its targets were decreased by hsa-miR-149-5p overexpression in a human lung epithelial cell line. Several studies, in different cancer contexts, support the oncogenic role of increased hsa-miR-149-5p (48). hsa-miR-149-5p is upregulated in non–small cell lung tumors compared with adjacent tissues (59), lower expression of hsa-miR-149-5p inhibited cell growth in prostate cancer cells *in vitro* (60), and higher exosomal hsa-miR-149-5p was associated with metastatic melanoma (61). Notably, Srivastava and colleagues (62) showed that IFNγ activity may suppress hsa-miR-149-5p levels and drive an inflammatory response in keratinocytes. We observed a similar association in which progressive/persistent proliferative PMLs have decreased expression of interferon signaling genes and high levels of hsa-miR-149-5p compared with regressive PMLs. Additionally, defects in antigen processing and presentation are hallmarks of cancer immune evasion (63). Many of the targets of hsa-miR-149-5p are inactivated by other oncogenic mechanisms to alter the antigen presentation activity in cancer cells, including the loss of heterozygosity of HLA and B2M genes (64, 65), somatic mutation in HLA genes (66), epigenetic alterations (67, 68), and transcriptional regulation (54). Less is known in bronchial PMLs; however, recent work showed that the loss of heterozygosity and hypermethylation in the HLA regions are associated with dysfunctional antigen processing and presentation in CIS lesions (11). Our findings suggest a posttranscriptional regulatory mechanism by which upregulation of hsa-miR-149-5p in airway basal cells impairs antigen processing and presentation, contributing to immune evasion.

Demonstrated by miR-ISH, hsa-miR-149-5p expression is increased in epithelial tissue regions compared with nonepithelial regions, and in regions of dysplasia, its expression is higher in progressive/persistent compared with regressive PMLs. The resolution of hsa-miR-149-5p expression to basal cells, however, was not definitive as we did not have a KRT5 counter stain. In the IMC data, we observed a significant decrease in NLRC5 expression in basal, basal/differentiating, KRT5-low basal, and MUC5B^+^ secretory cells in regions of hyper-metaplasia and dysplasia in progressive/persistent versus regressive PMLs. As hsa-miR-149-5p expression is assayed across the whole tissue via miR-ISH, but the IMC results are based on ROIs, direct integration of the results was not possible. The results, however, suggest that hsa-miR-149-5p and NLRC5 expression were anticorrelated in regions of dysplasia. Of the epithelial cell types with decreased NLRC5 expression associated with progression, basal cells were the only cell type in which the level of NLRC5 influenced its spatial proximity to CD8 T cells. These observations were consistent with *in vitro* experiments that showed decreased *NLRC5* expression that results in reduced MHC-I surface expression and CD8 T cell–mediated cytotoxicity. Basal cells are the progenitor stem cells and suggested to be the cell of origin of LUSC in the proximal airway (69, 70). Laughney and colleagues (71) demonstrated an inverse association between expression levels of SOX2 (a transcription factor specifying lung cell fate) and MHC-I genes in primary and metastatic lung tumors. Similarly, the stem cell program in colon cancer has been associated with decreased levels of antigen presentation (72). These observations suggest that the cell states of basal cells may be an important determinant of the immune surveillance and evasion in bronchial PML progression. We also observed that CEACAM5^+^ peri-goblet cells, a smoking-associated cell population (29), were enriched with increasing histologic severity and in progressive/persistent compared with regressive proliferative PMLs. We did not, however, detect a change in NLRC5 expression in these cells associated with progression/regression status or that NLRC5 levels influence their spatial proximity to CD8 T cells. Future investigations will be needed to further unravel the causality between epithelial cell state, expression of hsa-miR-149-5p and NLRC5, and the functional ability of cells to present antigen and recruit immune cells.

The IMC data also revealed that NLRC5 protein expression increases as the histology grade advances from normal epithelium to hyper-metaplasia and dysplasia accompanied by an increase in CD8 T cells, CD4 T cells, and NK cells. In progressive/persistent compared with regressive proliferative PMLs, there is a decrease in NLRC5 protein expression within hyper-metaplasia and dysplasia regions and CD8 T cells, B cells, and M2 macrophages are decreased. Spatial analysis demonstrated that NLRC5-high basal cells are in significantly closer proximity to CD8 T cells compared with NLRC5-low basal cells. Levels of NLRC5 were also shown to decrease in basal cell populations in areas of hyper-metaplasia and dysplasia in progressive/persistent compared with regressive proliferative PMLs. Similar relationships between NLRC5-regulated MHC-I gene expression and cancer immune evasion have been extensively studied in the context of cancer (53, 54). Using the solid tumor data from TCGA, Yoshihama and colleagues (73) demonstrated that lower NLRC5 expression is associated with deficient CD8 T-cell activation and poor prognosis. Furthermore, recent work demonstrated that impaired MHC-I processing and presentation pathway is related to resistance to immune therapy (74) and poor outcomes (75) in lung cancer, and increased MHC-I gene expression can improve immunotherapy response (76), suggesting their critical roles and therapeutic potential in the context of lung cancer management.

In summary, utilizing miRNA–gene network with miR-ISH and IMC assays to identify and resolve the spatial and cellular localization of hsa-miR-149-5p, its downstream targets, and the immune microenvironment, we have identified a posttranscriptional regulatory mechanism that may contribute to immune evasion and bronchial PML progression. Our results suggest cell type–specific biomarkers of lesion progression and a potential epithelial-specific candidate therapeutic target to potentially reverse early immune evasion. Future experiments in model systems are needed to unravel regulatory mechanisms of hsa-miR-149-5p, to confirm the extent to which hsa-miR-149-5p regulates NLRC5 levels and the immune microenvironment, and to understand the association between MHC-I gene expression and recruitment of cytotoxic CD8 T cells. Additionally, testing anti-miRNA oligonucleotides (77, 78) to target hsa-miR-149-5p in preclinical models, such as the NTCU mouse (79, 80), is needed to show their efficacy in preventing or delaying lesion progression. Uncovering potential regulators of early immune evasion holds promise to advance lung cancer chemoprevention in patients at high risk for developing lung cancer.

## Supplementary Material

Supplementary Tables 1-12 Supplementary Tables 1-12

Figure S1 Supplementary Figure S1. Correlation between GSVA scores of miRNAs and gene modules from the miRNA-gene network.

Figure S2 Supplementary Figure S2. Antigen presentation module genes were enriched among genes negatively correlated with hsa-miR-149-5p in lung-related datasets.

Figure S3 Supplementary Figure S3. Expression level of hsa-miR-149-5p was significantly negatively correlated with that of *NLRC5* in lung-related datasets.

Figure S4 Supplementary Figure S4. Expression of hsa-miR-149-5p was up-regulated and expression of *NLRC5* was downregulated in LUSC tumor compared to adjacent benign tissue.

Figure S5 Supplementary Figure S5. Flow cytometry experiment details.

Figure S6 Supplementary Figure S6. Correlation between hsa-miR-149-5p and *NLRC5* expression levels within the samples from the FANTOM5 project.

Figure S7 Supplementary Figure S7. Performance of the hsa-miR-149-5p spot detection classifier.

Figure S8 Supplementary Figure S8. hsa-miR-149-5p was associated with histology grade in progressive lesions.

Figure S9 Supplementary Figure S9. Identification of broad cell types in IMC data.

Figure S10 Supplementary Figure S10. Analysis of IMC data from the epithelial tissue.

Figure S11 Supplementary Figure S11. Analysis of IMC data from the non-epithelial tissue.

Figure S12 Supplementary Figure S12. Representative IMC images.

## Data Availability

For endobronchial biopsy and airway brushing miRNA expression data, raw FASTQ files and the miRNA expression matrices and associated sample and clinical data are available on Gene Expression Omnibus under accession number GSE313146 and the Human Tumor Atlas Network Data Portal (https://humantumoratlas.org/explore) using the identifications provided in Supplementary Table S5. For endobronchial biopsy imaging data, H&E, miR-ISH, and IMC images are available on Zenodo (81) and the miR-ISH and IMC images are also available via the Human Tumor Atlas Network Data Portal (https://humantumoratlas.org/explore) using the identifications provided in Supplementary Table S5. For endobronchial biopsy and airway brushing gene expression data, raw FASTQ files are available via dbGaP accession phs003185.v1.p1 and the gene expression count and residual matrices are available via Gene Expression Omnibus under accession number GSE109743. Gene expression residual data from GSE109743 were used to construct miRNA–mRNA correlation network (10). For additional publicly available endobronchial biopsy data, miRNA expression data from GSE93284 and gene expression from GSE66499 and GSE114489 were used to validate the miRNA–mRNA correlation and gene association with lesion outcomes (12, 50, 82). For *FANTOM5* gene and miRNA expression data, *FANTOM5* miRNA expression profiles for 2,595 mature miRNAs across 399 samples (directly downloaded from https://fantom.gsc.riken.jp/5/suppl/De_Rie_et_al_2017/in November 2022; ref. 30) were used. The library size–normalized (CPM) miRNA expression matrix was log_2_-transformed with pseudocount of 0.1. Matched gene expression profiles in transcripts per million [(TPM); *N* = 394) were downloaded from the same website and were used to examine the miRNA–mRNA correlation by cell type. The tag count of the most supported peak (p1@NLRC5) was used for the expression of *NLRC5* per sample. The TPM values were log_2_-transformed with pseudocount of 0.1 for correlation analysis. For TCGA-LUSC mRNA and miRNA expression data, sample-matched gene and miRNA expression data for the TCGA LUSC (*N* = 475) were also used to validate the miRNA–mRNA correlation. We also examined differences between has-miR-149-5p and *NLRC5* in matched TCGA LUSC tumor tissue and adjacent benign tissue (*n* = 45 paired samples for miRNA and *n* = 51 paired samples for mRNA). Gene (legacy) and miRNA expression data of the primary tumor samples were obtained using TCGAbiolink (49) in October 2022. Counts associated with the same mature miRNA transcript were aggregated. miRNA count normalization was performed as described above. The Pearson correlation coefficients were then calculated between miRNA and mRNA data on expression residuals adjusting for the plate as previously described (10). For gene and miRNA expression from bronchial brushings from the AEGIS trials, gene expression and miRNA expression data from sample-matched main stem bronchus brushing samples (*N* = 341) were obtained from AEGIS clinical trials. Gene-level expression data were obtained from GSE66499 (51) and were processed as previously described (83). miRNA-seq data were obtained from GSE93284 (50). For the mRNA–miRNA network construction, three miRNA target databases were used. For TargetScan v7.2 (24), all conserved miRNA sites were downloaded from targetscan.org. miRNAs with an additional suffix (0.1 or 0.2) compared with miRbase were both included. For starBase (25), all predictions for hg19 were downloaded from starbase.sysu.edu.cn on December 2022 using the API and were filtered for clipExpNum ≥ 5. For miRTarBase v7.0 (26), the  HUGO Gene Nomenclature Committee (HGNC) symbols were translated to Ensembl IDs (GRCh37) using biomaRt (84). All code is available via the following Github repository, https://github.com/jbeane0428/miR149.git.
