# Supplementary material for: Upregulation of an Epithelial miRNA Is Associated with Immune Evasion in Progressive Bronchial Premalignant Lesions
Source: Cancer Immunol Res. 2026 Feb 11;14(4):689–707. doi: 10.1158/2326-6066.CIR-25-0431 (PMC12969512; doi:10.1158/2326-6066.CIR-25-0431)
Supplement: Figure S1 — Supplementary Figure S1. Correlation between GSVA scores of miRNAs and gene modules from the miRNA-gene network. [file cir-25-0431_figure_s1_supps1.pdf]

Supplementary Figure S1

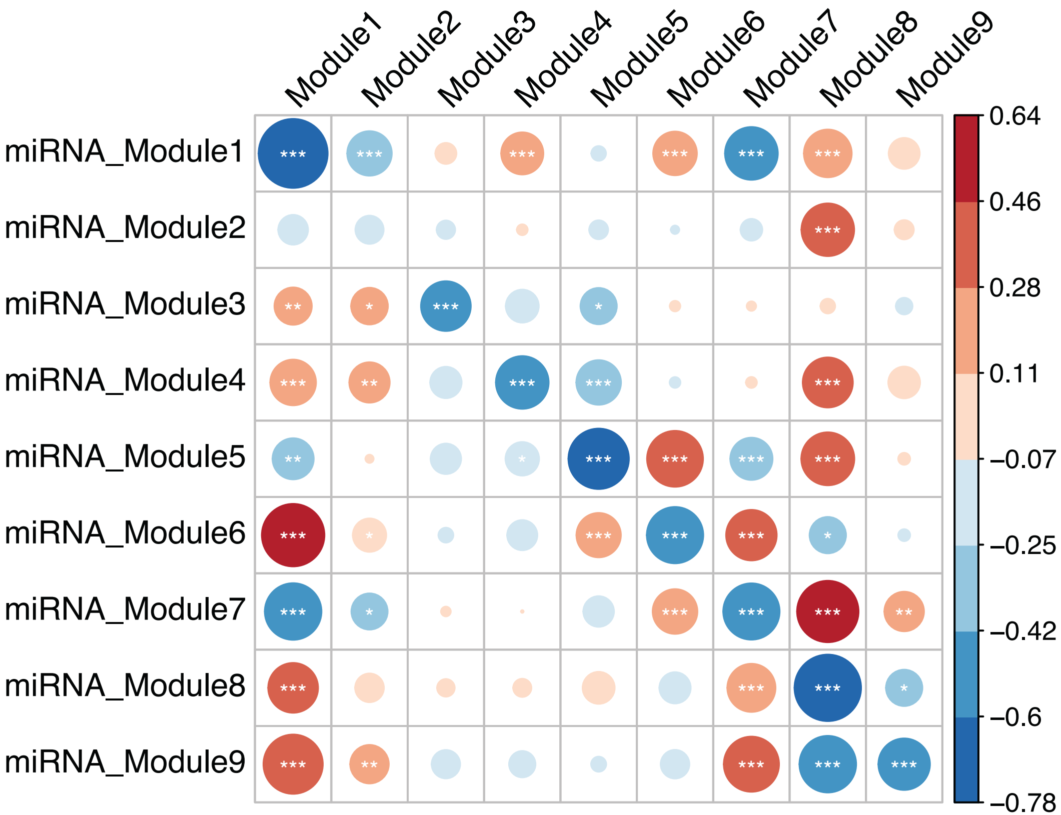

Supplementary Figure S1. Correlation between GSVA scores of miRNAs and gene modules from the miRNA-gene network. Bubble plots of the Pearson correlation between GSVA scores of module associated miRNAs and gene modules from the miRNA-gene module network. Each miRNA connected to predicted target genes within a gene module and that has passed the statistical tests outlined in the methods was assigned to that gene module. The expression value of all miRNAs assigned to a gene module was calculated using GSVA. * FDR <= 0.05; ** FDR <= 0.01; *** FDR <= 0.001.
