# Supplementary material for: Upregulation of an Epithelial miRNA Is Associated with Immune Evasion in Progressive Bronchial Premalignant Lesions
Source: Cancer Immunol Res. 2026 Feb 11;14(4):689–707. doi: 10.1158/2326-6066.CIR-25-0431 (PMC12969512; doi:10.1158/2326-6066.CIR-25-0431)
Supplement: Figure S2 — Supplementary Figure S2. Antigen presentation module genes were enriched among genes negatively correlated with hsa-miR-149-5p in lung-related datasets. [file cir-25-0431_figure_s2_supps2.pdf]

Supplementary Figure S2

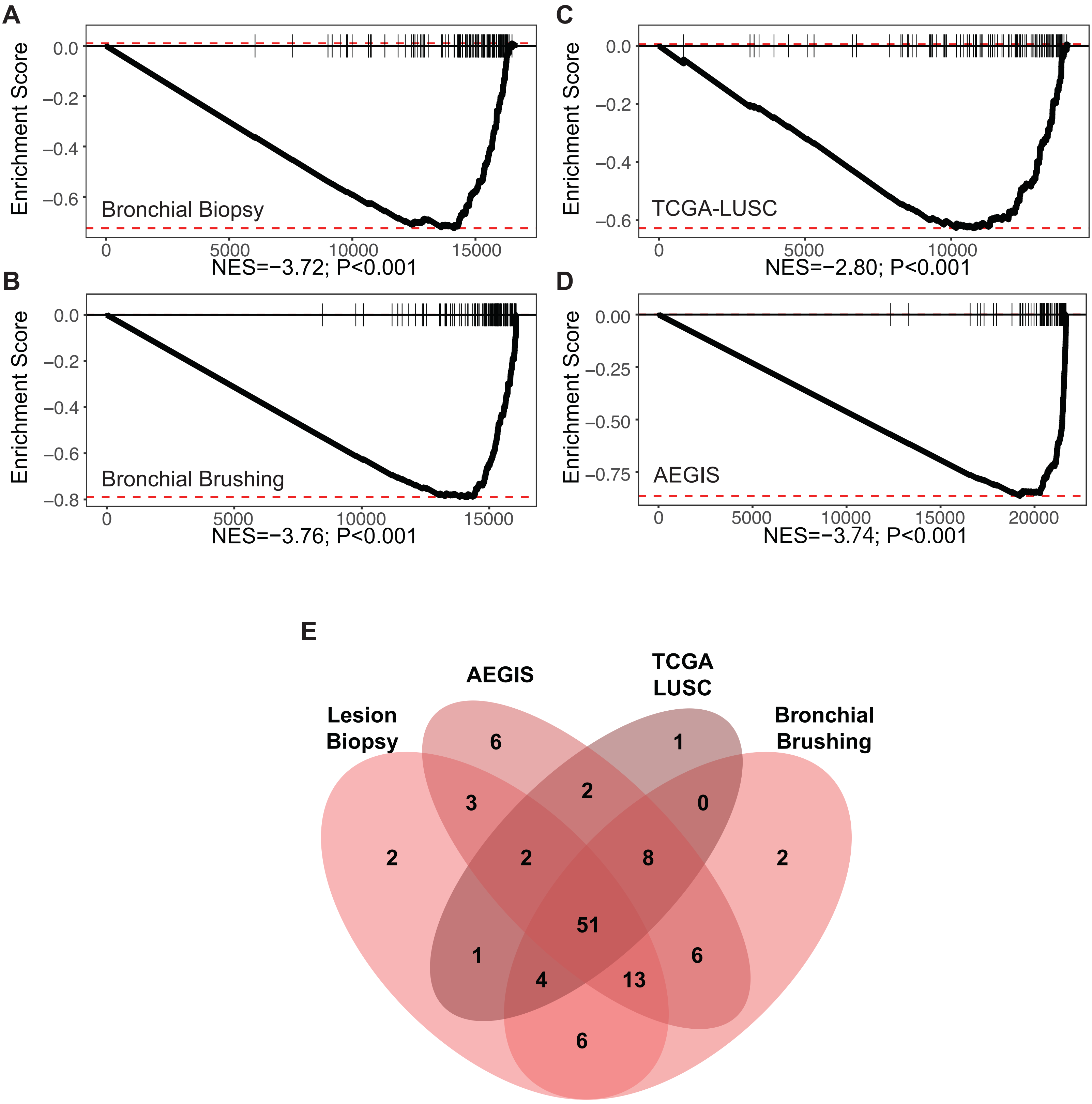

Supplementary Figure S2. Antigen presentation module genes are enriched among genes negatively correlated with hsa-miR-149-5p in lung-related datasets. Enrichment plot of module 9 genes (n=112) among all genes ranked by their expression level correlation with hsa-miR-149-5p across four datasets: (**A**) biopsy samples (n=156), (**B**) bronchial brushing samples (n=87) from this study, (**C**) TCGA-LUSC primary tumor samples (n=475), (**D**) AEGIS bronchial brushing samples (n=341). (**E**) Overlap of leading-edge genes from analyses in **A-D**.
