# Supplementary material for: Upregulation of an Epithelial miRNA Is Associated with Immune Evasion in Progressive Bronchial Premalignant Lesions
Source: Cancer Immunol Res. 2026 Feb 11;14(4):689–707. doi: 10.1158/2326-6066.CIR-25-0431 (PMC12969512; doi:10.1158/2326-6066.CIR-25-0431)
Supplement: Figure S3 — Supplementary Figure S3. Expression level of hsa-miR-149-5p was significantly negatively correlated with that of NLRC5 in lung-related datasets. [file cir-25-0431_figure_s3_supps3.pdf]

Supplementary Figure S3

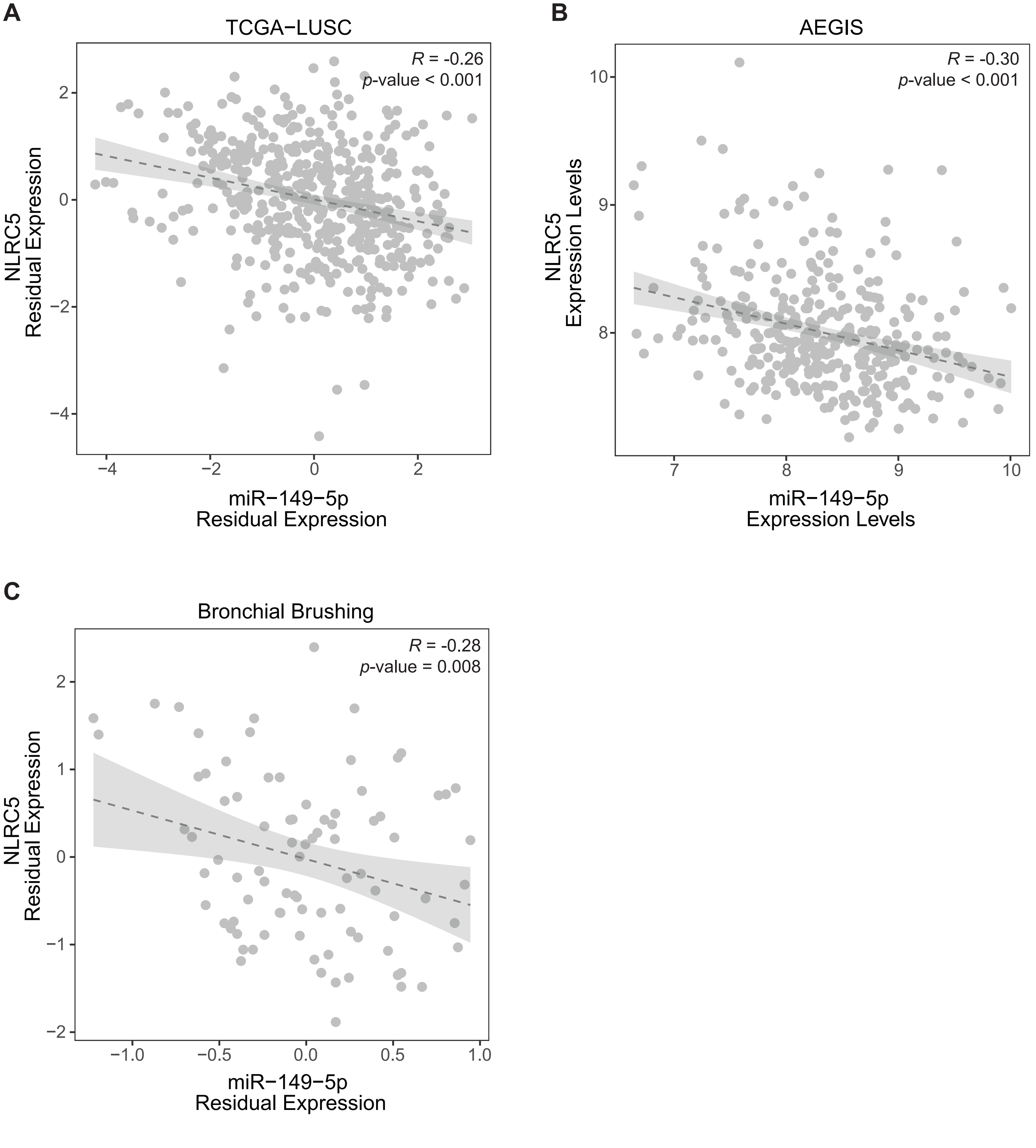

Supplementary Figure S3. Expression level of hsa-miR-149-5p was significantly negatively correlated with that of NLRC5 in lung-related datasets. Scatterplots showing the Pearson correlation between the expression levels of hsa-miR-149-5p and NLRC5 across three datasets: (**A**) TCGA-LUSC primary tumor samples (n=475), (**B**) AEGIS bronchial brushing samples (n=341), and (**C**) bronchial brushing samples (n=87) from this study. The dashed line represents the linear regression fit and the shaded gray region indicates the 95% confidence interval. There was a significant negative correlation, calculated using Pearson correlation, between hsa-miR-149-5p and NRLC5 in all datasets (**A-C**).
