# Supplementary material for: Upregulation of an Epithelial miRNA Is Associated with Immune Evasion in Progressive Bronchial Premalignant Lesions
Source: Cancer Immunol Res. 2026 Feb 11;14(4):689–707. doi: 10.1158/2326-6066.CIR-25-0431 (PMC12969512; doi:10.1158/2326-6066.CIR-25-0431)
Supplement: Figure S4 — Supplementary Figure S4. Expression of hsa-miR-149-5p was up-regulated and expression of NLRC5 was downregulated in LUSC tumor compared to adjacent benign tissue. [file cir-25-0431_figure_s4_supps4.pdf]

Supplementary Figure S4

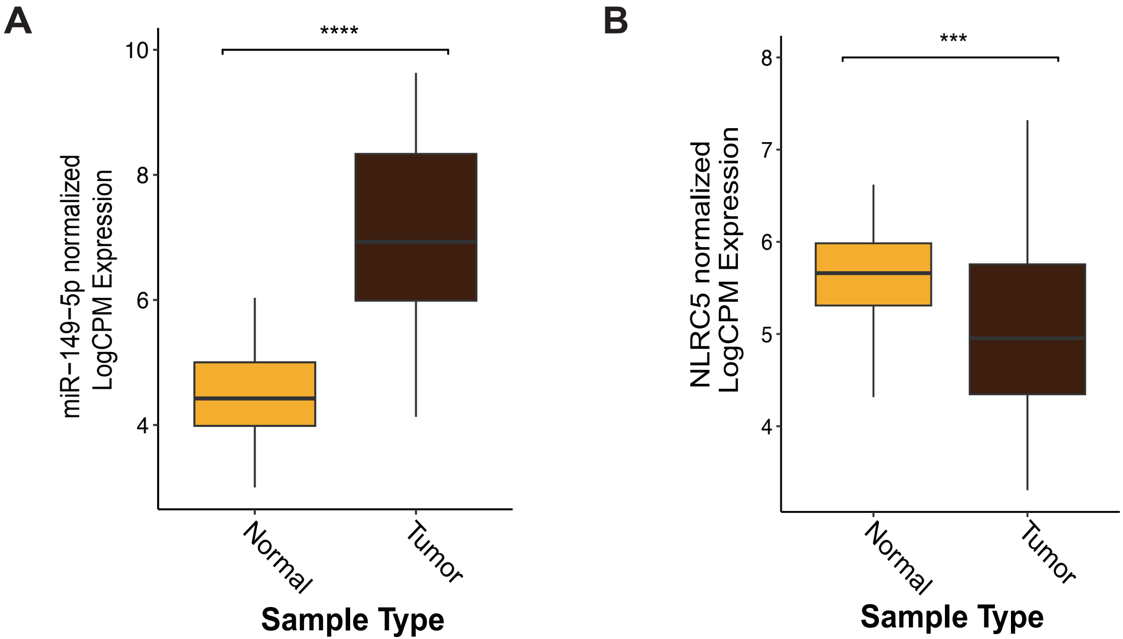

Supplementary Figure S4. Expression of hsa-miR-149-5p was up-regulated and expression of NLRC5 was downregulated in LUSC tumor compared to adjacent benign tissue. Boxplots show normalized TCGA LUSC logCPM levels of (**A**) hsa-miR-149-5p (n = 45 pairs) and (**B**) NLRC5 (n = 51 pairs) between adjacent benign tissue (yellow) and LUSC tumor tissue (dark red). Data indicate median with IQR, and whiskers indicate minimum and maximum measurement. P values were determined by linear mixed effect models. ***P <= 0.001, ****P <= 0.0001
