# Supplementary material for: Upregulation of an Epithelial miRNA Is Associated with Immune Evasion in Progressive Bronchial Premalignant Lesions
Source: Cancer Immunol Res. 2026 Feb 11;14(4):689–707. doi: 10.1158/2326-6066.CIR-25-0431 (PMC12969512; doi:10.1158/2326-6066.CIR-25-0431)
Supplement: Figure S5 — Supplementary Figure S5. Flow cytometry experiment details. [file cir-25-0431_figure_s5_supps5.pdf]

Supplementary Figure S5

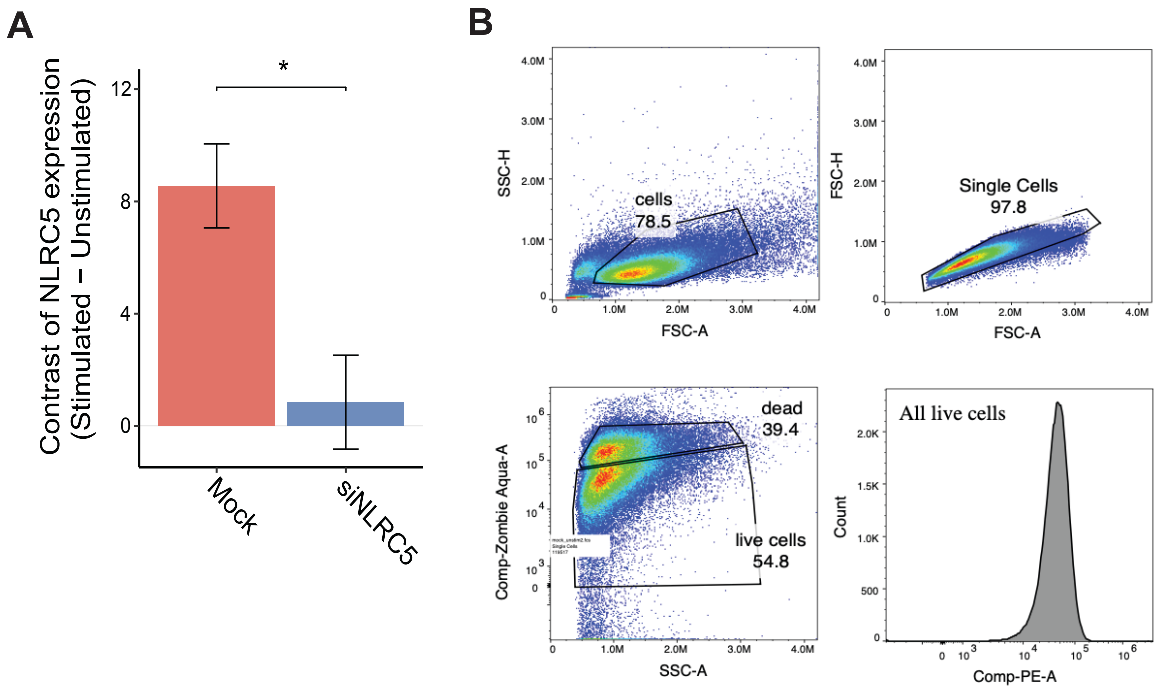

Supplementary Figure S5. Flow cytometry experiment details. (**A**) Barplot showing the difference in NLRC5 expression detected by qRT-PCR between IFN-r simulated and unstimulated conditions within mock transfected (n=3) or siNLRC5 transfected (n=3) SW900 cells. The expression level of NLRC5 is decreased after IFN-r stimulation in the siNLRC5 transfected cells compared to mock transfected cells. P-value is determined by liner model. Error bars indicate standard errors. *P<= 0.05. (**B**) Cells were initially gated based on FSC-A versus SSC-H, followed by a gate on FSC-A versus FSC-H to select the single cells. Live and dead cells were discriminated by Zombie Aqua and live cells were used for downstream analysis.
