# Supplementary material for: Upregulation of an Epithelial miRNA Is Associated with Immune Evasion in Progressive Bronchial Premalignant Lesions
Source: Cancer Immunol Res. 2026 Feb 11;14(4):689–707. doi: 10.1158/2326-6066.CIR-25-0431 (PMC12969512; doi:10.1158/2326-6066.CIR-25-0431)
Supplement: Figure S6 — Supplementary Figure S6. Correlation between hsa-miR-149-5p and NLRC5 expression levels within the samples from the FANTOM5 project. [file cir-25-0431_figure_s6_supps6.pdf]

Supplementary Figure S6

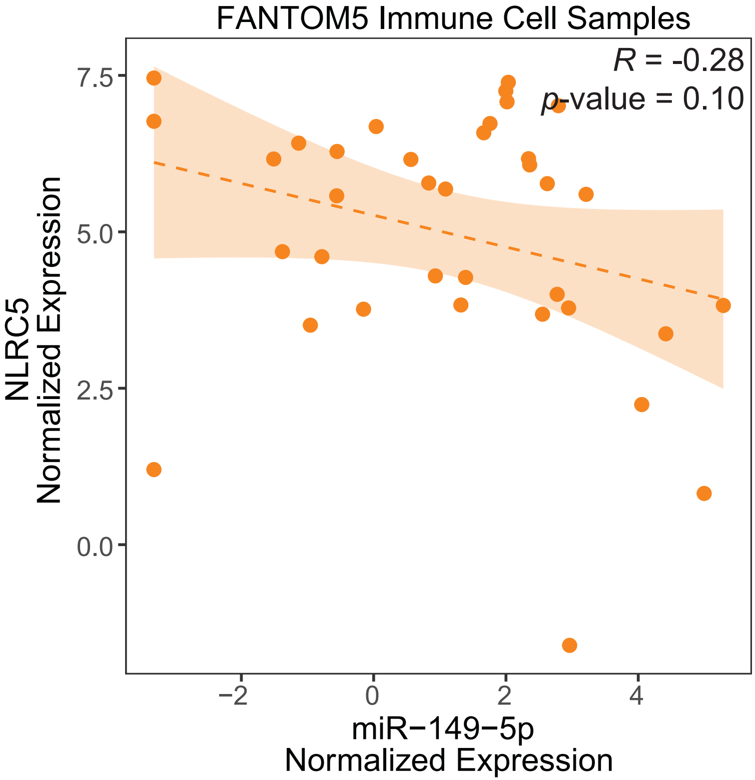

Supplementary Figure S6. Correlation between hsa-miR-149-5p and NLRC5 expression levels within the samples from the FANTOM5 project. Scatter plot of the Pearson correlation between the normalized expression levels of hsa-miR-149-5p and NLRC5 within the FANTOM5 samples derived from immune (n=37) cell compartments. The orange dashed line represents the linear regression fit and the shaded region indicates the 95% confidence interval.
