# Supplementary material for: Upregulation of an Epithelial miRNA Is Associated with Immune Evasion in Progressive Bronchial Premalignant Lesions
Source: Cancer Immunol Res. 2026 Feb 11;14(4):689–707. doi: 10.1158/2326-6066.CIR-25-0431 (PMC12969512; doi:10.1158/2326-6066.CIR-25-0431)
Supplement: Figure S7 — Supplementary Figure S7. Performance of the hsa-miR-149-5p spot detection classifier. [file cir-25-0431_figure_s7_supps7.pdf]

Supplementary Figure S7

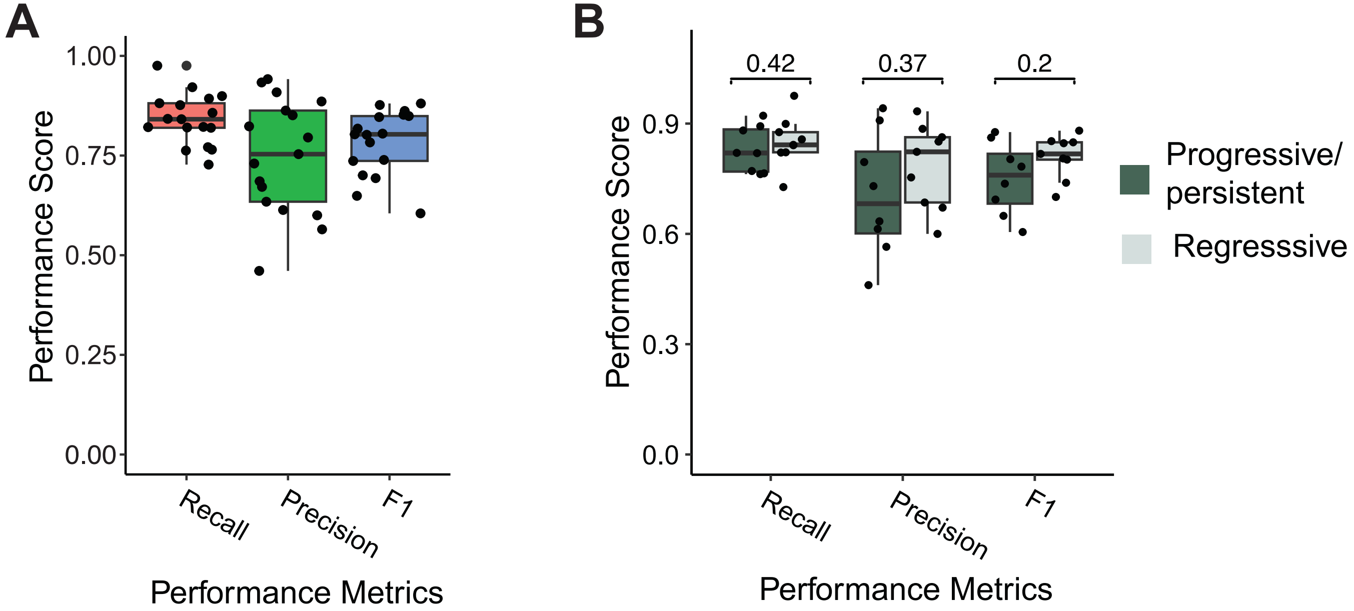

Supplementary Figure S7. Performance of the hsa-miR-149-5p spot detection classifier. (**A**) Boxplot showing the recall (red orange), precision (green), and F1(blue) scores for hsa-miR-149-5p detection classifier on regions in each miR-ISH image containing manual spot annotations (n = 19). (**B**) Boxplot showing the recall, precision and F1 scores for the hsa-miR-149-5p detection classifier between progressive/persistent PMLs (dark green, n= 10) and regressive PMLs (light green, n = 9). There are no significant differences in the performance metrics between progressive/persistent PMLs and regressive PMLs. Data indicate median with IQR, and whiskers indicate minimum and maximum measurement.
