# Supplementary material for: Upregulation of an Epithelial miRNA Is Associated with Immune Evasion in Progressive Bronchial Premalignant Lesions
Source: Cancer Immunol Res. 2026 Feb 11;14(4):689–707. doi: 10.1158/2326-6066.CIR-25-0431 (PMC12969512; doi:10.1158/2326-6066.CIR-25-0431)
Supplement: Figure S8 — Supplementary Figure S8. hsa-miR-149-5p was associated with histology grade in progressive lesions. [file cir-25-0431_figure_s8_supps8.pdf]

Supplementary Figure S8

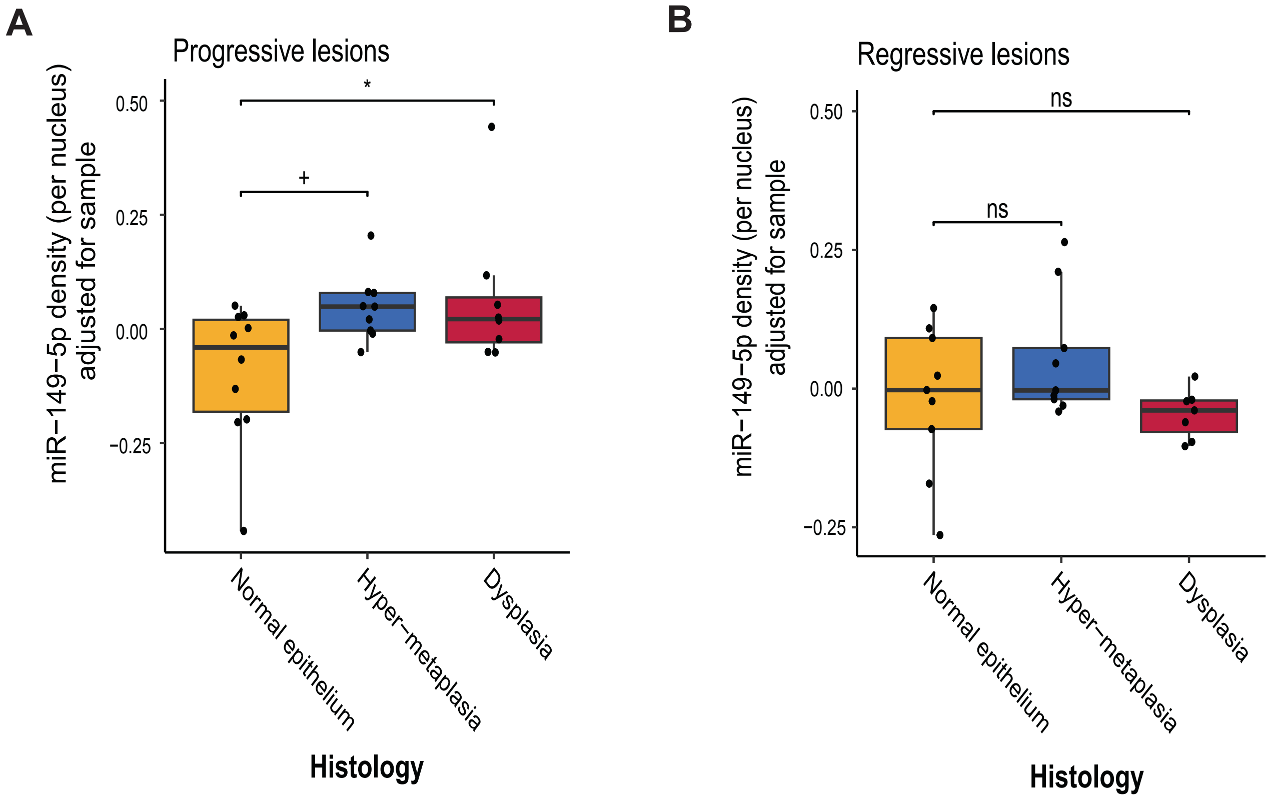

Supplementary Figure S8. hsa-miR-149-5p is associated with histology grade in progressive lesions. Boxplots showing hsa-miR-149-5p spot density in dysplastic regions was increased compared to normal epithelium in (**A**) progressive, but not in (**B**) regressive lesion. Data indicate median with IQR, and whiskers indicate minimum and maximum measurement. P values were determined by linear mixed effect models. +P <= 0.1, *P <= 0.05, **P <= 0.01, ***P <= 0.001; ns, no significance
