# Supplementary material for: Upregulation of an Epithelial miRNA Is Associated with Immune Evasion in Progressive Bronchial Premalignant Lesions
Source: Cancer Immunol Res. 2026 Feb 11;14(4):689–707. doi: 10.1158/2326-6066.CIR-25-0431 (PMC12969512; doi:10.1158/2326-6066.CIR-25-0431)
Supplement: Figure S9 — Supplementary Figure S9. Identification of broad cell types in IMC data. [file cir-25-0431_figure_s9_supps9.pdf]

Supplementary Figure S9

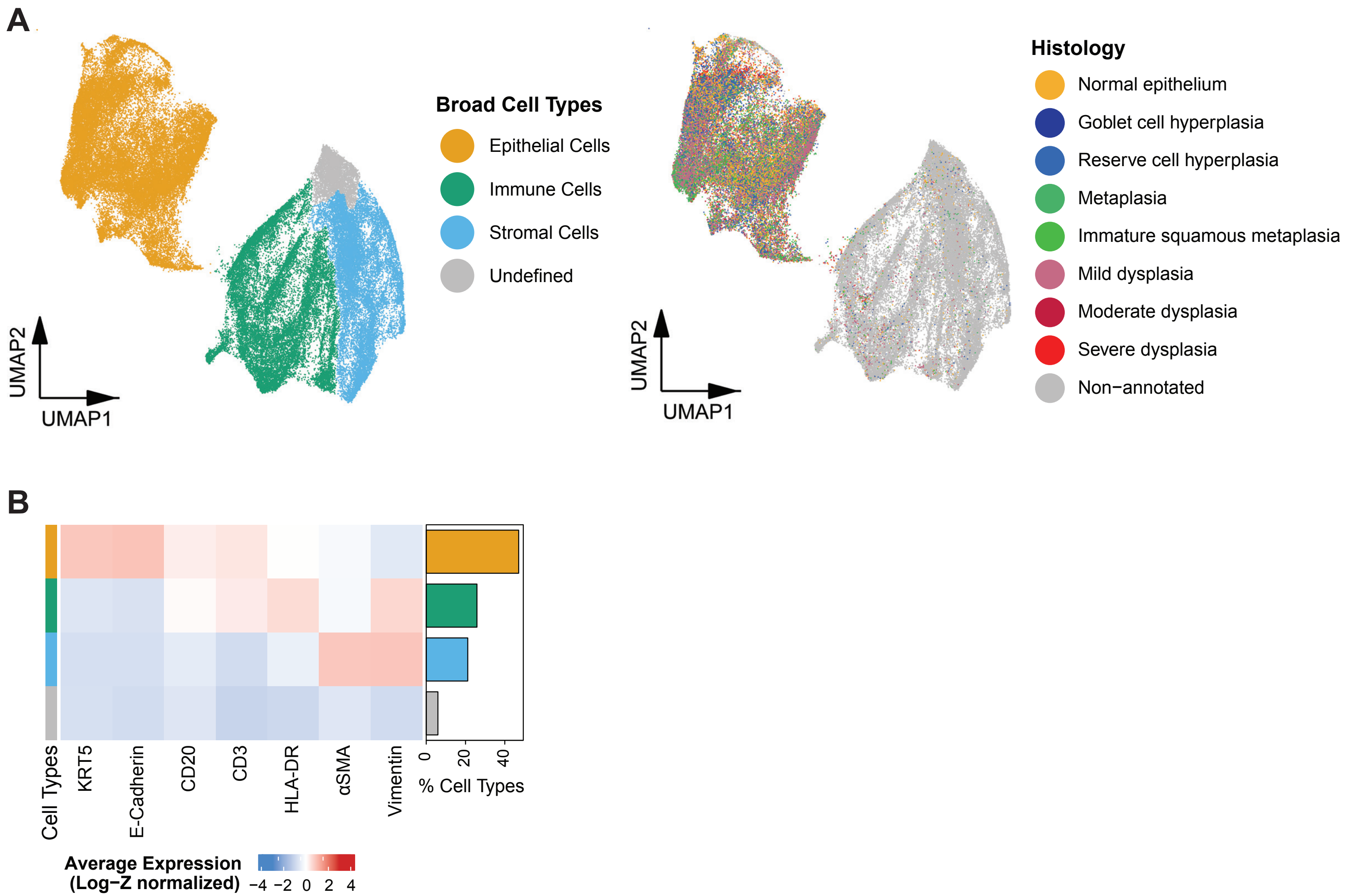

Supplementary Figure S9. Identification of broad cell types in IMC data. (A) Uniform Manifold Approximation and Projection (UMAP) plots showing 3 broad cell types (left) and histology annotations (middle) for all cells in the IMC images (n = 87,401). **(B)** Heatmap showing the average expression of canonical markers across the broad cell types with the bar plot showing the relative proportion of each cell type.
