# Supplementary material for: Upregulation of an Epithelial miRNA Is Associated with Immune Evasion in Progressive Bronchial Premalignant Lesions
Source: Cancer Immunol Res. 2026 Feb 11;14(4):689–707. doi: 10.1158/2326-6066.CIR-25-0431 (PMC12969512; doi:10.1158/2326-6066.CIR-25-0431)
Supplement: Figure S10 — Supplementary Figure S10. Analysis of IMC data from the epithelial tissue. [file cir-25-0431_figure_s10_supps10.pdf]

Supplementary Figure S10

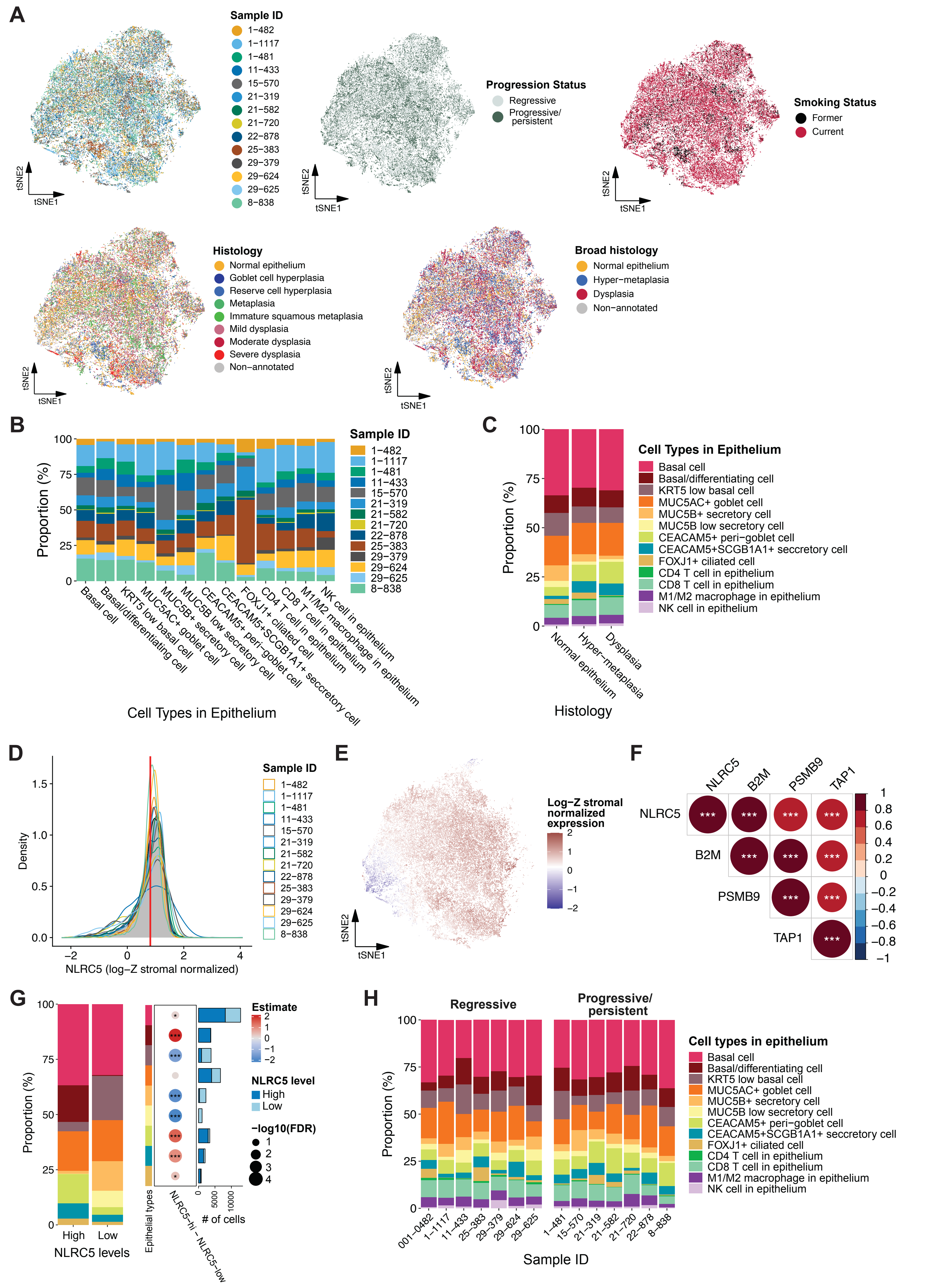

Supplementary Figure S10. Analysis of IMC data from the epithelial tissue. (**A**) tSNE visualization of cells within the epithelium (n = 41,147 cells) colored by sample ID (left top), progression status (middle top), smoking status (right top), histology (left bottom), and histology groups (middle bottom). (**B**) Stacked bar plot showing the relative proportion of cells contributed by each sample for each cell type identified within the epithelium. (**C**) Stacked bar plot showing the relative proportion of cell types across different histology groups in epithelium. (**D**) Density plots showing the distribution of the log-Z stromal normalized NLRC5 expression across epithelial cell populations for each sample. The gray shade shows the distribution of values across all samples, and the red line represents the average of this distribution. The red line was used to dichotomize the cells within the epithelium into NLRC5-hi or NLRC5-low cells. (**E**) tSNE plot of cells within the epithelium colored by log-Z stromal normalized NLRC5 expression. (**F**) Bubble plot showing the correlation between NLRC5 expression and the expression of its downstream targets in epithelial cells. Color and dot size represent the Pearson correlation coefficient. (**G**) Stacked bar plot showing the relative proportions of epithelial cell types across NLRC5-high or NLRC5-low epithelial cells (left). Bubble plot showing the differential composition of 9 epithelial cell types between NLRC5-high and NLRC5-low epithelial cells. Bar plot to the right of the dot plot shows the number of cells in each cell type stratified by NLRC5-high versus low expression. Dot color represents the compositional estimate, and dot size represents the log FDR value computed by sccomp. (**H**) Stacked bar plot showing the relative proportion of cell types identified in epithelium for each sample stratified by PML outcome (regressive versus progressive/persistent). P values were FDR adjusted and determined by the t-test for correlation significance for (**F**), and the sccomp differential composition test for (**G**). * P <= 0.05, **P <= 0.01, ***P <= 0.001.
