# Supplementary material for: Upregulation of an Epithelial miRNA Is Associated with Immune Evasion in Progressive Bronchial Premalignant Lesions
Source: Cancer Immunol Res. 2026 Feb 11;14(4):689–707. doi: 10.1158/2326-6066.CIR-25-0431 (PMC12969512; doi:10.1158/2326-6066.CIR-25-0431)
Supplement: Figure S11 — Supplementary Figure S11. Analysis of IMC data from the non-epithelial tissue. [file cir-25-0431_figure_s11_supps11.pdf]

Supplementary Figure S11

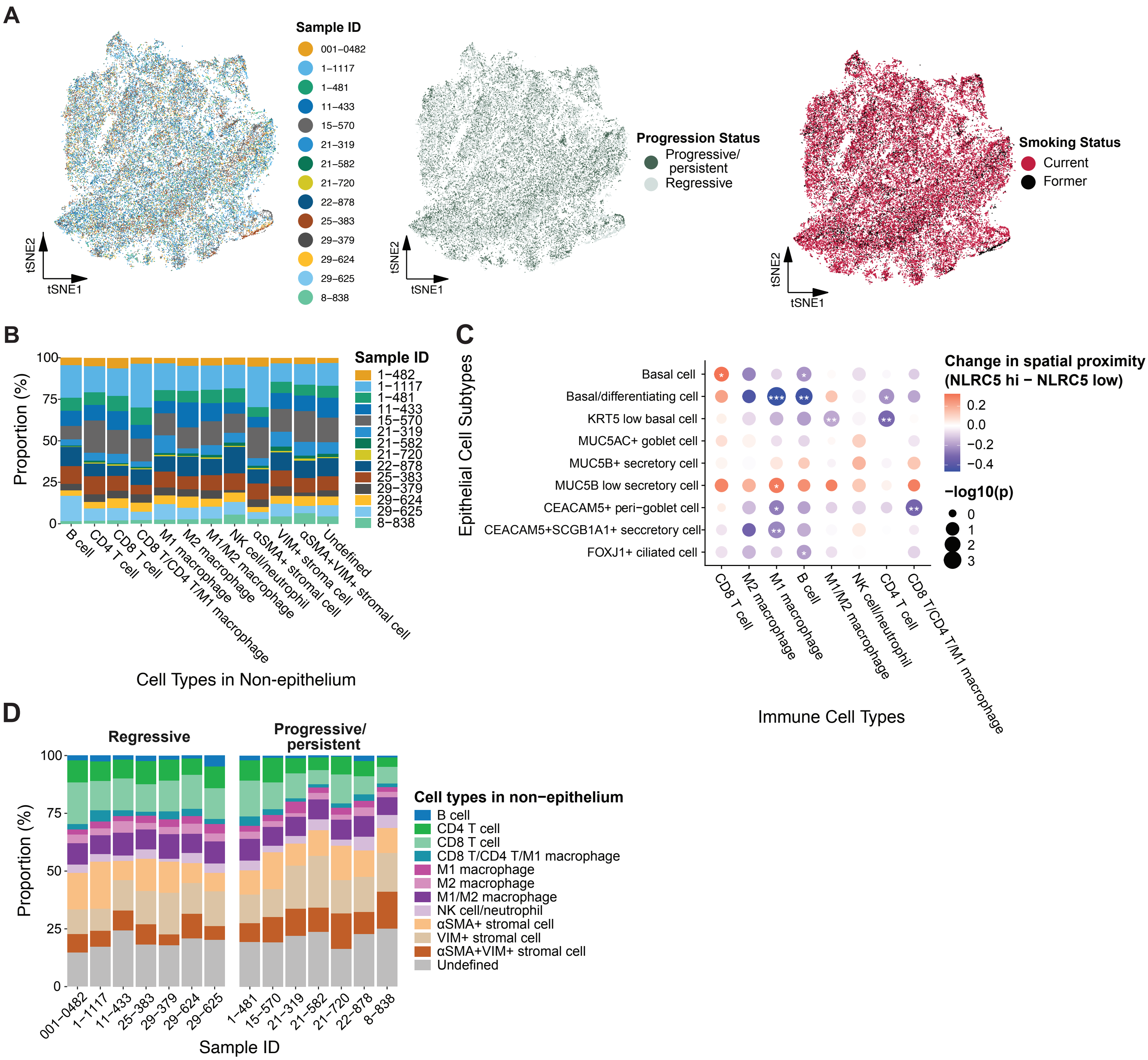

Supplementary Figure S11. Analysis of IMC data from the non-epithelial tissue. (**A**) tSNE visualization of cells not in epithelium (n = 46,254 cells) colored by sample ID (left), progression status (middle), and smoking status (right). (**B**) Stacked bar plot showing the relative proportion of cells contributed by each sample for each cell type identified within non-epithelium. (**C**) Bubble plot showing for each epithelial cell type the change in spatial proximity between NLRC5-high and NLRC5-low cells and immune cell populations. Dot color represents the changes in spatial proximity where red indicates closer spatial proximity between an immune cell population and NLRC5-high compared to NLRC5-low cells of a specific epithelial cell type, and dot size represents the log p-value. P values were determined by the two-sided paired Wilcoxon test where asterisks denote * P <= 0.05, **P <= 0.01, ***P <= 0.001. (**D)** Stacked bar plot showing the relative proportion of cell types identified in the non-epithelium for each sample stratified by PML outcome (regressive versus progressive/persistent).
