# Supplementary material for: Upregulation of an Epithelial miRNA Is Associated with Immune Evasion in Progressive Bronchial Premalignant Lesions
Source: Cancer Immunol Res. 2026 Feb 11;14(4):689–707. doi: 10.1158/2326-6066.CIR-25-0431 (PMC12969512; doi:10.1158/2326-6066.CIR-25-0431)
Supplement: Figure S12 — Supplementary Figure S12. Representative IMC images. [file cir-25-0431_figure_s12_supps12.pdf]

Supplementary Figure S12

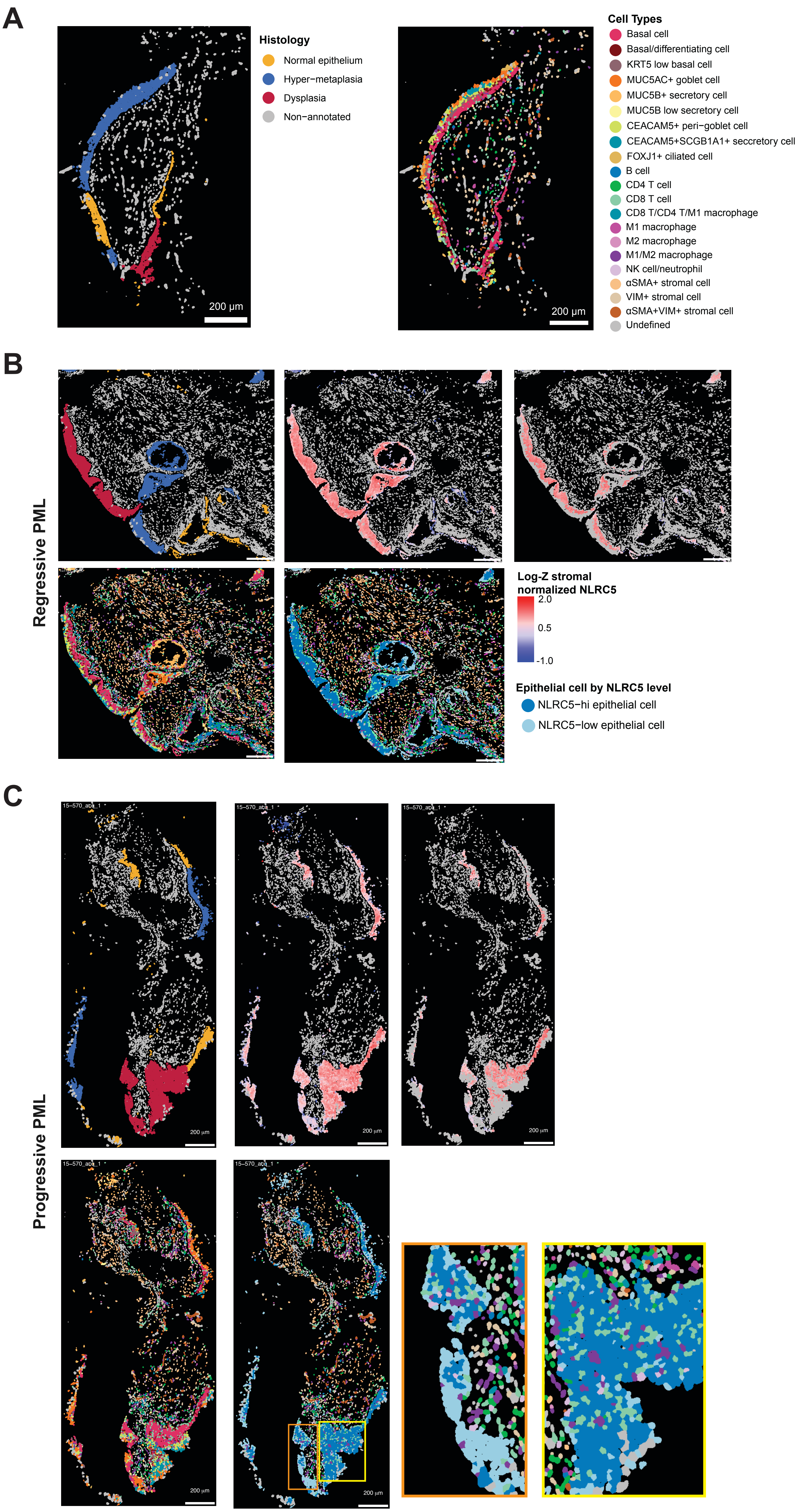

Supplementary Figure S12. Representative IMC images. (A) Representative IMC image where the segmented cells are shown colored by histology group (left) and cell type (right). (**B** and **C**) Representative IMC images of a progressive PML (**B**) and a regressive PML (**C**) where segmented cells are colored by histology group (top left), NLRC5 expression of epithelial cells (top middle), NLRC5 expression of basal cells (top right), cell types (bottom left), and spatial proximity of NLRC5-high versus NLRC5-low cells with immune cells (bottom middle). In (**C**) the yellow and orange boxes in the bottom middle figure denote regions that are shown at a higher magnification.
